# Talking trash: Perspectives on community environmental health in the Dominican Republic

**DOI:** 10.1371/journal.pone.0248843

**Published:** 2021-03-29

**Authors:** Chloe Turner, Maura A. Powell, Rodney R. Finalle, Kate Westmoreland, Kevin Osterhoudt, Ramona Cordero Paulino, Elizabeth D. Lowenthal

**Affiliations:** 1 Department of Pediatrics (Global Health), The Children’s Hospital of Philadelphia, Philadelphia, Pennsylvania, United States of America; 2 Departments of Pediatrics and Biostatistics, Epidemiology and Informatics, University of Pennsylvania Perelman School of Medicine, Philadelphia, Pennsylvania, United States of America; 3 Niños Primeros en Salud Program (Affiliate of The Children’s Hospital of Philadelphia), Consuelo, Dominican Republic; Helen Keller International, SIERRA LEONE

## Abstract

A safe and healthy natural and built environment is fundamental to children’s health and represents a significant determinant of community well-being. We aimed to identify and prioritize environmental health concerns within resource-poor neighborhoods in the Dominican Republic using free-listing and semi-structured focus groups composed of parents and caregivers in the perirural community of Consuelo, Dominican Republic. Transcripts were coded and relevant themes identified using qualitative content analysis. Demographic data and information regarding trash disposal practices were also collected. Participants described common health concerns, including respiratory infections, asthma, vector-borne illnesses, and diarrheal diseases and linked them to environmental hazards in their communities, such as air quality and sanitation. Interventional priorities that emerged included reduction of trash accumulation and trash burning as well as improvement of sanitation facilities.

## Introduction

A safe and healthy environment is necessary to promote and protect children’s health and community well-being [1]. Globally, ensuring environmental sustainability has been prioritized as one of the eight United Nations Millennium Development Goals and is now included in the Sustainable Development Goals [2]. Poverty and environmental threats are closely linked, and resource-limited settings bear a disproportionate burden of child morbidity and mortality linked to modifiable environmental health factors [3].

Environmental risks contribute to a large fraction of the global burden of disease. It is estimated that 24% of global deaths and 28% of deaths among children under five are due to preventable environment-related causes, including respiratory infections, diarrheal illnesses, and vector-borne diseases [4]. Children are especially vulnerable to environmental exposures due to factors related to their growth, development, and behavior [4–6]. Environmental hazards in low- and middle-income countries, including indoor and outdoor air pollution, water contamination, and poor sanitation, have detrimental impacts on the well-being of children. Reducing environmental risks is vital to improving children’s health. A systematic review of health research on children exposed to environmental pollutants in Latin America showed the need for more research to guide pediatric practice, advocacy, and public policy [7].

At 28.8 per 1000, the mortality rate for children under 5 years of age in the Dominican Republic (DR) ranks 64^th^ in the world, and 30% of the population lives below the poverty line [8–10]. In high-poverty communities in the DR, the solid waste management systems are inadequate and fragmented [11]. Access to public and private garbage collection services is often sporadic and varies between and within municipalities. With little recycling available, much of the country’s trash ends up in one of about 350 open-air landfills and improvised dump sites are common across the country, often near populated areas [12, 13]. In poorer areas, waste often accumulates on roads, empty lots, and in bodies of water, posing a public health threat to residents [14, 15]. In July 2018, the DR made international headlines when waves of trash washed ashore on beaches near the capital city of Santo Domingo; over sixty tons of garbage were collected from the shore in one week [16]. While it is estimated that 85% of the population in urban areas uses at least basic sanitation services, defined as facilities that ensure hygienic separation of human excreta from human contact, including toilets or latrines not shared with other households, sanitation services are more limited in rural areas [17].

National regulations seeking to improve waste separation and promotion of recycling were proposed by the Ministry of Environment in 2018 [12, 18, 19]. Locally in some areas of the country, community-based initiatives have shown success with improving environmental conditions through emphasis on separating waste in schools through composting and neighborhood clean-up efforts [12]. In the perirural municipality of Consuelo, DR, community members have voiced concern regarding the health effects on young children related to contaminated water, air pollution, and solid waste management practices, including litter accumulation and trash burning in the town’s poorest neighborhoods. For the community to successfully work towards advancing child health in the long term, it is fundamental to systematically identify and understand the community’s environmental health priorities and vision in order to make the case for change and initiate feasible interventions [20].

The primary objective of this study was to identify and prioritize environmental health concerns as they relate to children’s health among families living in the most impoverished neighborhoods in Consuelo, DR. The secondary objective was to describe their vision of potential community-based efforts that are likely to lead to health improvements through environmental change. This project aims to inform resource allocation to advance long-term environmental health in this community, using approaches designed to be generalizable to other similar communities.

## Methods

### Setting and participants

The study was reviewed by the Children’s Hospital of Philadelphia Institutional Review Board on July 01, 2014, and the IRB determined that it is exempt from IRB review. CONABIOS (Consejo Nacional de Bioética en Salud), the National Commission on Bioethics in Health in the Dominican Republic, agreed with this determination. All applicable national regulations and laws applying to both local and foreign investigators were followed.

Participants included parents and caregivers (18 years of age or older) of children receiving health services from the Niños Primeros en Salud (NPS) primary care pediatric clinic in Consuelo, DR, a perirural municipality inland in the southeast region with a population of about 30,000 people, surrounded by sugar cane fields [21]. The NPS program cares for over 400 children younger than 5 years of age who live in six barrios (neighborhoods) that were chosen for proximity to the clinic and severity of poverty. These barrios are similar to many impoverished communities in the DR.

### Design

We used a combination of purposive and convenience sampling to recruit parents and caregivers of young children. We purposely selected caregivers who serve as community health workers (“promotoras”) in their barrios, given their demonstrated passion for community engagement and child health promotion. Additional parents were invited to participate during routine clinic and home visits during the study time frame with the goal of balancing enrollment from the different barrios within the clinic’s catchment area.

The study was conducted between August and October 2014. The verbal free-listing exercise asked participants to independently identify and list concerns regarding children’s health and environmental issues in their barrio. The data obtained from the individual free-listing activity was used to guide facilitator probes during the focus group discussions (FGD). FGDs were directed with a semi-structured discussion guide focused around (1) describing health issues that impact children in each barrio, (2) discussing environmental concerns observed in each barrio that are linked to these health issues, and (3) identifying potential community-based interventional opportunities to address the issues.

FGDs were held in each of the six barrios, and each involved 6–10 participants. The lead investigator facilitated the groups in Spanish, and each FGD lasted between 40 minutes and 1 hour. Each participant also completed an individual verbal questionnaire providing information about personal demographics, family composition, length of time they have lived in the barrio, household trash disposal practices, and type of sanitation facility used by household members. The focus groups were audio recorded with an assistant taking notes (e.g. who was speaking when they didn’t identify themselves verbally). Audio recordings were transcribed verbatim.

Relevant themes were identified using qualitative content analysis. Two study investigators reviewed all transcripts and agreed on the preliminary coding structure based on emergent themes regarding environmental health priorities and the community’s vision of community-based efforts that are likely to effectively lead to health improvements through environmental change. The preliminary coding structure was organized into major themes, reviewed, and enhanced by other team members. Two study investigators independently coded each of the transcripts. Coding discrepancies were resolved through discussions between the coders, with assistance from a third researcher when needed. Salient quotes were translated into English for inclusion in this manuscript.

## Results

Characteristics of the 55 participants are presented in Table 1. Most were females who had lived in the community for more than 10 years. Table 2 illustrates the household trash disposal practices reported by participants. Many participants reported that their families use more than one method of trash disposal, depending on type of trash and reliability of the municipal trash collection. Eighty-four percent of participants reported that they participate in some sort of municipal trash collection service and 42% of participants reported burning trash. About half of participants (49%) reported using a family-owned latrine or toilet facility outside the home, while 35% reported using community/shared latrines. Fewer than 20% of participants had an indoor toilet with plumbing.

**Table 1:** Participant characteristics.

Characteristics	Participants
***Total Number of Participants***	55
***Gender–N (%)***	
Female	50 (91)
Male	5 (9)
***Age***	
Median (IQR) in years	30 (22–36)
***Ethnicity–N (%)***	
Dominican	43 (78)
Haitian	7 (13)
Haitian-Dominican	5 (9)
***Employment Status—N (%)***	
Not Employed	34 (61)
Fixed Employment	13 (24)
Per-Diem Position	8 (15)
***Education Completed—N (%)***	
No Schooling	1 (2)
Primary School	13 (24)
Middle School	20 (36)
High School	21 (38)
***Marital Status—N (%)***	
Single	24 (43)
Cohabiting/Married	29 (53)
Widowed	1 (2)
Separated/Divorced	1 (2)
***Number of Children in Household***	
Median (IQR)	3 (2–4)
***Time Lived in Neighborhood—N (%)***	
Less than 1 year	1 (2)
Between 1–5 years	9 (16)
Between 6–10 years	16 (29)
More than 10 years	29 (53)

**Table 2:** Trash disposal practices of participants.

Type of Trash Disposal Practice	Participants
3 or More Means of Trash Disposal	17 (31%)
Municipal Trash Collection Only	15 (27%)
Municipal Trash Collection AND Food Scraps for Animals	14 (26%)
Municipal Trash Collection AND Trash Burning	4 (7%)
Trash Burning AND Food Scraps for Animals	4 (7%)
Trash Burning Only	1 (2%)
*Total*	*55 (100%)*

### Environmental impact on health: Community concerns

The most common child health concerns identified by participants through the free-listing exercise were:

1. Respiratory infections and asthma
2. Vector-borne illnesses
3. Diarrheal/gastrointestinal diseases

Participants endorsed beliefs that their child health concerns are frequently related to environmental hazards in their communities. Top environmental concerns identified by participants through the free-listing exercise were:

1. Air pollution, primarily related to trash burning
2. Inadequate sanitation facilities
3. Trash accumulation

Details related to each of these environmental health concerns are outlined below.

#### 1. Air pollution and respiratory concerns

Trash burning was identified as the main contributor to poor air quality. Participants described trash burning as a common practice, often necessitated by limitations of the municipal trash collection system (e.g. infrequent and unreliable garbage pick-ups, broken garbage collection trucks, limited access in some of the poorest neighborhoods). Given the proximity of houses to one another, participants were concerned about the effects of neighbors burning trash. One mother of three children described why many families resort to trash burning:

*The garbage truck does not come often to collect the trash…people reach a state of exasperation and they say, “Well, I have this trash pile in my yard that I can’t stand,” so they decide to burn it…and if they burn trash right here, even though I live in that house over there, the smoke affects my children*.

Concerns about trash burning were amplified in a barrio that is adjacent to the town dump. Municipal garbage trucks bring the collected trash to the dump and trash is often burned there. Residents of the adjacent community report that they see and smell smoke from the trash pile on most days. A mother who resides in this barrio expressed concerns regarding the relationship of asthma and trash burning:

*Asthma is a problem because the town’s garbage dump is very close. We breathe all the smoke from the trash that is burned there, and many children then have trouble breathing. Those of us adults that have respiratory or heart problems are also affected. At night, you can feel the smoke burning your throat*.

Many participants voiced unease about what kind of trash material was burned (including plastic and tire rubber) and the health effects that may have on people in the community. One mother described her situation:

*Trash burning happens commonly here. Once when I was pregnant and they were burning trash, I had to leave my house because I could barely breathe and was suffocating. It seemed like they were burning something toxic that was harming me*.

#### 2. Sanitation and diseases

Another major theme that arose from discussions was the state of sanitation facilities and water quality and the associated adverse health effects on the community, including gastrointestinal illnesses and mosquito-borne diseases. One participant summed up her concerns as:

*Sometimes there is no safe water, and children drink contaminated water and become sick with amoebas*.

Water is often collected from neighborhood water spigots. These are sometimes located close to sanitation facilities. Participants voiced concern that the spouts often leak, leading to pools of standing water, which can become breeding grounds for mosquitoes.

*Regarding the spigots, there is one that has very green water and many mosquitoes. A mosquito bit my nephew and he had dengue, and I think it came from that water source. The spigots attract many mosquitoes*.

Concerns were raised regarding inadequate sanitation/toilet facilities. Shared latrines are commonly located outside of homes. In some areas, it is difficult to avoid contact with human excreta. Some participants described safety concerns with sending their children outside to toilet facilities at night and reported sometimes using plastic bags or buckets that were then deposited in fields or trash piles. One participant described:

*Where I live, there are people who defecate in a plastic bag and then toss it wherever they want. I once found a plastic bag of feces on the roof of my house—it smelled terrible*.

While the above anecdote was not a routine community practice, disposal of human waste in fields, ravines, or abandoned lots was reported as common. Disposal of human waste outside of latrines was sometimes necessitated by inadequate numbers of available sanitation facilities. For example, one participant described having only four toilets for an area where over 50 people live. Challenges to building more sanitation facilities include physical space constraints near already crowded homes and economic constraints. Many families rent their houses from landlords who they felt were not responsive to their concerns and requests for improved facilities.

#### 3. Trash accumulation and health effects

Focus group participants related the limited access to municipal trash collection services to subsequent trash accumulation in neighborhood streets, paths, and abandoned lots. Trash piles seemed to attract cockroaches, mice, stray dogs, and mosquitoes. One mother described:

*The garbage truck hardly ever comes to collect trash. Sometimes it does not come to this barrio for months, and for that reason a lot of garbage accumulates here. When trash accumulates, it produces mosquitoes because the trash piles fill with water*.

Another participant described:

*There are many mice…and the trash piles attract more mice*.

A father of three children similarly expressed concerns regarding trash piles with standing water and the association with mosquito breeding areas:

*In the road, those discarded bottles and cans can fill up with water, and mosquitoes begin to breed. The accumulated trash brings mosquitoes—it is a pile of diseases*.

Some of the barrios are located next to large sugar cane fields. Participants described how many people dispose of trash and human waste in the tall sugar cane stalks when the trash collection truck does not come for a few weeks at a time:

*The garbage truck sometimes does not come. So, people throw trash in the sugarcane field and that causes problems with mosquitoes and pollution*.

### Community-based interventional opportunities

Participants identified potential community-based interventions to address some of the neighborhood environmental concerns and children’s health. The most common identified opportunities were:

1. Neighborhood education initiatives
2. Reusing/repurposing materials to reduce trash accumulation
3. Partnering with existing community/neighborhood groups to advocate for change
4. Group clean-up activities
5. Approaching authority figures regarding community matters related to environmental health

Details regarding each of these community-based interventional opportunities are outlined below.

#### 1. Neighborhood education initiatives

Discussion among participants revolved around ways to increase knowledge about health and the environment among community members. Participants acknowledged a need for increasing neighborhood awareness regarding environmental health as it relates to children. As one father stated:

*Guidance and training [regarding environmental health] should be given regularly like medicine so that everyone can understand…and so they don’t forget*.

Participants described how neighbors’ actions and practices affect those around them, often causing frustration, and cited the need for improved communication between neighbors and community-wide action:

*There are ways for us to improve our environment, but it doesn’t help to just do this in our own houses. We should join together and talk with our neighbors so we can spread the word. There are times when I clean my yard; I don’t throw garbage on the ground; I don’t have standing water. But my neighbor’s yard does have this, and the rats and cockroaches go from one yard to another*.

#### 2. Repurposing reusable materials

Some participants voiced interest in learning how to make crafts from used items instead of throwing them away in an effort to reduce trash accumulation. Participants discussed selling these crafts and creating an economic incentive to use repurposed items instead of discarding them in the trash. Other participants agreed that this would be feasible and desirable since many caregivers are unemployed and looking for ways to use their time productively. As one mother described:

*With used plastic bottles and jars, people can make beautiful things, and I would like to learn to do that. Then we can sell things and make some money*.

#### 3. Partnering with existing groups

Focus group participants mentioned existing community groups and discussed the challenges and benefits of organizing the community to make change happen. One mother suggested:

*We could form a committee and go with (the president of the neighborhood council) to knock on doors—if one person goes, no one will listen, but if a group of 10 or 15 people go, then people will pay attention and we can resolve things more quickly*.

#### 4. Group clean-up

During some focus groups, participants discussed how neighborhood clean-up days have been successful in the past, and described how the community could organize more of these:

*We can talk with all our neighbors and one Saturday or Sunday start to clean up together and weed together*.

Another participant enthusiastically agreed:

Ok, let’s do it! Everyone to work! Women with their brooms and men with their machetes!

#### 5. Approaching authority figures

Participants discussed challenges related to approaching community leaders and governmental organizations regarding neighborhood matters, including infrequent municipal trash collection service. Multiple participants expressed frustration that they did not trust elected officials to be their advocates. However, they did see this as a potential way for community members to work together to address issues. As one mother articulated:

*All the mothers…can go directly to the town hall to talk with the leaders and let them know that we have the right…for the trash to be cleaned up. The city council can come to the neighborhood and see for themselves*.

Others expressed that problems between renters and landlords are barriers to improved sanitation. One participant stated:

*The people that live here should demand that the landlords know that the renters need a toilet, and without that, they are causing harm. …If the landlords do not see interest and if they get paid every month and nobody says anything, the landlords will not do anything*.

## Discussion

It is estimated that nearly 100,000 children <5 years of age in the Americas die each year from physical, biological, and chemical hazards in the environment [22]. Children living in Latin America and the Caribbean have long faced a complex array of environmental threats to health related to hazards of air pollution, contaminated drinking water, and solid waste accumulation, as well as newer environmental threats such as pesticides, heavy metals including lead and mercury, hazardous waste including electronic waste, and climate change driven by urbanization. The assortment of environmental hazards and the health risks they cause varies among Latin American countries and regionally within countries, depending on social determinants of health, demographics, and urbanization [23]. Although these health issues are magnified for young children, they are important to people of all ages.

In 2015, the DR Environment and Natural Resources Minister cautioned that the country could “be in a state of permanent vulnerability due to deficient waste collection and disposal.” [24] Participants in this study underscored this vulnerability as they talked about their own utilization of multiple trash disposal practices due to sporadic access to municipal waste management services. Recent DR Ministry of Environment proposals focus on improving waste separation, and policy change is imperative to help mitigate environmental health threats [12]. Though this study took place in 2014, the situation remains similar and the findings unfortunately are still relevant. This study can help to guide next steps in the Consuelo community with the renewed energy of this new legal framework.

Community-based initiatives in other regions of the DR have shown promise in efforts to clean up informal dumping sites in neighborhoods and nearby bodies of water, to educate students about inorganic/organic waste separation by implementing composting in schools, and to support more formal recycling programs [12]. To plan effective community-based interventions, communities must identify local priorities as well as potential initiatives that seem feasible, as this study accomplishes. The results of this study can directly inform planning, development, and allocation of resources to address the priorities identified by caregivers of children in Consuelo, DR. Unfortunately, since this study was conducted, other priorities such as the COVID-19 pandemic have made it difficult to advance issues related to environmental health. The need is now greater.

Although the sample size for this study was relatively small, consistent themes were raised between all 6 communities. The study provides insight into concerns about impacts of environmental hazards on children’s health from the unique perspective of parents and caregivers of young children, who are known to be the most vulnerable to the impacts of environmental hazards. Strengths of this study include recruitment of stakeholders based on their common roles as caregivers for young children, discussions held in “safe” settings where participants could speak openly and confidentially, and the use of multiple researchers to observe, transcribe, code, and analyze data to help ensure that perspectives were accurately captured. Focusing the discussions around child health outcomes helped to motivate participants since children are valued within these communities. Caregivers’ energy and motivation in the context of child protection could be leveraged to enact change in these and other communities where child health issues related to environmental hazards are recognized.

## Conclusion

Environmental health hazards prioritized by parents of young children in the DR include air pollution from trash burning, poor sanitation, and health effects of trash accumulation. Respiratory illnesses, diarrheal diseases and vector-borne diseases are thought to be common sequelae of these hazards. Practical approaches to address these concerns were proposed. This research demonstrates how community engagement can inform future community-based environmental health interventions centered around improving pediatric health outcomes in the Caribbean and Latin America.

## Supporting information

S1 File (DOCX)

S2 File (DOCX)

S3 File (DOCX)

S4 File (DOCX)

S5 File (DOCX)

S6 File (DOCX)

S7 File (DOCX)

S8 File (DOCX)
