# Supplementary material for: Talking trash: Perspectives on community environmental health in the Dominican Republic
Source: PLoS One. 2021 Mar 29;16(3):e0248843. doi: 10.1371/journal.pone.0248843 (PMC8007031; doi:10.1371/journal.pone.0248843)
Supplement: S1 File — (DOCX) [file pone.0248843.s001.docx]

# Focus Group Discussion Guide Script (English)

Talking Trash: Perspectives on Community Environmental Health in the Dominican Republic

**Introduction**

Welcome. Thank you for attending today’s focus group. *(Brief introduction of group leader and assistant.)*

During this focus group, I will ask questions and help facilitate conversation. Please keep in mind that there are no right or wrong answers to any of the questions. The purpose is to stimulate conversation and hear the opinions of everyone in the room. I hope you will be comfortable speaking honestly and sharing your ideas with us. Please note that it is *very important* to refrain from repeating or discussing comments made by people here outside of the group. This is to make sure that everyone can feel comfortable expressing anything that they think is important, even if what they want to say is sensitive.

Everyone’s ideas are important so please give everyone an opportunity to speak. The most important rule is that only one person speaks at a time. You do not have to speak in any particular order. You do not have to agree with the views of other people in the group. The reason we are meeting together to discuss this is to get many different opinions and to explore them in depth. We expect the focus group to last about 60 minutes, but it may take more time depending on how many ideas everyone wants to share.

Please remember that this session will be recorded with two audio recorders and my assistant will take some notes to ensure that we adequately capture your ideas during the conversation. Do you have any questions before we begin?

Let’s do a quick round of introductions. Can each of you tell the group your name and how many children live in your household? *(Note: Names will be changed to numbers in the transcript.)*

**Discussion**

- **Health**

You all shared your opinions during the free listing exercise about what the most important health issues are in your barrio. I will read the list of your ideas, ranked in order of frequency in the free listing exercise. Let’s discuss these ideas. *(The list will be on the white board, but I will read everything out loud.)*

- Which health issues are most common?
- Which health issues are most severe? (maybe affecting fewer children, but being a large burden to those families)
- Let’s hear why you each prioritized the health issues that you voiced individually and rank/vote on their order of importance together.
- **Environment**

You also shared your opinions about the environment in your barrio and how the physical surroundings and conditions of our homes, barrio, and shared community space affect the people living there. Here is your list of the most important issues in the barrio’s environment that affects children’s health, ranked in order of the frequency of the free listing exercise. Let’s discuss these ideas. *(The list will be on the white board, but I will read everything out loud.)*

- Which environmental problems impact health most?
- Which environmental problems could we most easily try to improve in order to improve health?
- **Future Directions**

Keeping in mind these health and environmental priorities, let’s discuss how the community move forward to address these problems, individually and collectively.

- What are some potential interventions for the problems you identified as most important?

*Prompts*:

- - How can the community work together to improve the environment of the barrio and make the barrio better for children’s health?
- If you could design a way to improve the environment in your barrio, what would it look like?
  - What are some specific ways to motivate individuals/mobilize the community to improve or change X?
  - What community-based efforts might work in this barrio to address these issues?
  - What can families and individuals in the barrio do to improve the environmental health for the future of the children here?
- Which of these interventions listed are most feasible?

*Prompts:*

- Which of these ideas are most likely to make a difference for the barrio?

**Concluding Question**

- Is there anything else that we haven’t discussed yet that you think is important to know about the health of children and families in this barrio and the relation to the barrio environment?

Thank you so much for participating. Your opinions are very valuable. We appreciate your time and hope that you have found the discussion interesting. I would like to remind you that any comments made during this discussion will be anonymous. Once again, please do not discuss comments made by individuals during this focus group outside of this room.
