# Supplementary material for: Talking trash: Perspectives on community environmental health in the Dominican Republic
Source: PLoS One. 2021 Mar 29;16(3):e0248843. doi: 10.1371/journal.pone.0248843 (PMC8007031; doi:10.1371/journal.pone.0248843)
Supplement: S2 File — (DOCX) [file pone.0248843.s002.docx]

# Free Listing Exercise Form (English)

Talking Trash: Perspectives on Community Environmental Health in the Dominican Republic

**Study Identification Number: ________ Barrio Code: ________ Date: ________**

I. Please think about your barrio and your household. I would like to hear about the health issues that affect the families (and especially the children) of this barrio. What are the most important health problems that affect children in this barrio? (Please tell me at least the three most important ones.)

| **Response** | **Comments** |
| --- | --- |
| 1. |  |
| 2. |  |
| 3. |  |
| 4. |  |
| 5. |  |

II. Now let’s think about health in relation to the barrio’s environment. The environment is the air, the water, the land, the houses and buildings, and everything in the community space that have an effect on the people who live here. What are the main issues or problems in the environment of your barrio that affect children’s health? Please tell me the most important issues in the barrio’s environment that can impact the health of children in this community. (Please tell me at least the three most important ones.)

| **Response** | **Comments** |
| --- | --- |
| 1. |  |
| 2. |  |
| 3. |  |
| 4. |  |
| 5. |  |
