# Supplementary material for: Talking trash: Perspectives on community environmental health in the Dominican Republic
Source: PLoS One. 2021 Mar 29;16(3):e0248843. doi: 10.1371/journal.pone.0248843 (PMC8007031; doi:10.1371/journal.pone.0248843)
Supplement: S3 File — (DOCX) [file pone.0248843.s003.docx]

**(*Introducción*)…**

**…Ustedes compartieron sus opiniones sobre problemas de salud más comunes… (*Explicación*)**

1. **La gripe**
2. **La fiebre**
3. **Amebas**
4. **Asma o dificultad para respirar**
5. **Diarrea/Vómitos**
6. **Dengue**

**Vamos a discutir en grupo. ¿Cuáles son los problemas más comunes que afectan a los niños en este barrio?**

-Las amebas.

**Háblanos más de eso. ¿Por qué es más común en su opinión?**

-Por los niños que comen muchas cosas dulces, y andan descalzos y juegan con la tierra, y eso.

**¿Más ideas?**

-Y la gripe también. Los niños de por acá sufren de mucha gripe también. Se enferman mucho por la gripe porque hay mucho polvo aquí en este barrio.

**¿Hay mucha gripe en este barrio?**

-Sí, aquí hay mucho polvo, mucha basura, y por eso le da más gripe al niño.

-Y el asma también, porque el basurero lo tenemos muy cerca, y entonces uno respira todo ese humo de la basura que la queman, y eso entonces se aprietan mucho los niños y nosotros los que tenemos problemas respiratorios y los que tenemos problemas del corazón también nos afecta. De noche uno siente ese humo que le pica en la garganta y le molesta.

-También, la diarrea y los vómitos porque a los niños les gusta estar mucho en el suelo y se ponen cosas en la boca y por eso le dan. Y le hace daño y luego viene la pupú, diarrea y eso, los vómitos, y beben agua y por eso viene todo eso.

-A parte de eso, también los mosquitos, que los mosquitos también dan fiebre y también le producen vómitos. Hay mucha basura cerca de la casa y agua apozada.

-También el dengue que por eso la gente dicen que cuando tienen algo atrás de la casa, como las aguas de la botellas, si tienen agua limpia que no la están utilizando hay que botarla para no haya dengue.

-Y hay que echarle cloro al agua.

-Sí.

**Hay algunos de estos problemas que no son tan comunes que la gripe tal vez, pero son más severos ósea cuando pasan son problemas más grandes para la familia?**

-Bueno, la chinkungunya que fue el más fuerte en ese momento pero vendrá una más fuerte que ese que no recuerdo el nombre exactamente.

-¿Qué viene?

-Ébola

-Dios no va a permitir que venga algo así.

- Esa dique mata ya se han muerto mucha gente.

**¿Pero no tenemos ebola aquí, verdad?**

-No gracias a Dios.

-Eso ni lo mencionen, pero va a llegar si Jesucristo quiere que llegue ella va a llegar.

-La gripe que cuando da después pasa a dar fiebre es porque hay infección.

**Entonces yo he escuchado de la gripe y de la dificultad para respirar que es lo que causa muchos problemas aquí. He escuchado que mencionaron diarrea y vómitos que a veces tiene que ver con el agua. También mencionaron amebas que tiene que ver con el agua, y enfermedades de mosquitos como dengue y chinkungunya.**

-Y la neumonía también tiene que ver con agua.

**Entonces no sé si podemos estar de acuerdo de dos o tres más importante en este barrio a veces es importante hacer un grupo para estar de acuerdo ¿cuál es problemas más grande de la salud de los niños aquí?**

-La gripe.

-Eso es los más grande, lo más grande. Lo que más afecta es la gripe y la fiebre porque cuando llega la gripe, siempre tiende a dar fiebre porque es que llega a una etapa que viene la infección, al niño mío cuando le da gripe, de una vez le ataca la neumonía, como que se le tapan los pulmoncitos y ahí viene. Lo atrapa la fiebre y tengo que internarlo. Entonces yo trato siempre de que no le de gripe, pero cuando se arma el brote siempre le da y eso es lo que más afecta.

**Gracias. ¿Hay otras opiniones? ¿Hay personas que piensen que tal vez hay algo más importante?**

-La diarrea, eso es por las cosas que agarran en el suelo, si encuentran algo sucio en el suelo ello lo agarran y se lo llevan a la boca y ya eso le produce diarrea, porque eso le va bajando a los intestinos. De ahí también se van enfermando y le da fiebre también.

-Sí, y también se van debilitando, poniéndose flaquito por los vómitos y después no tienen deseos de comer, y ahí se van poniendo flaco y flaco hasta que pierden peso.

-Yo entiendo que muchas cosas de esas pasan también.

-Los niños bajan de peso también y lo llevan al médico cuando están bajo de peso.

-Porque vivimos en un mundo donde estamos todos juntos vivimos la gente y los animales estamos todos juntos.

-Los niños se desnutren mucho también.

-Por los vómitos y la diarrea.

-Sí y bajan de peso.

**¿Y eso tiene que ver con qué? ¿Que causa ese problema?**

-Con la alimentación también tiene que ver. Los niños bajo en peso y viendo como los niños lo alimentan, porque no es solo es la diarrea, también si tú a un niño en vez de darle su comida que lleva a las doce, su desayuno, le das galleticas y cositas así y no lo alimentan bien se ponen bajo de peso.

-Pero la mayoría de las enfermedades que están en el barrio es por culpa de la basura porque está alrededor siempre. Los barrios siempre son así, cuando están descuidados y la basura uno la tira donde quiera y comen cualquier cosa, que un pote lo tiran donde quiera y de ahí se llena de agua y de ahí viene la gripe, la fiebre, el asma todo eso.

-Respecto a la desnutrición yo diría: que eso no tiene nada que ver con la otra, si un niño cuando le da la diarrea y le da los vómitos si puede caer en desnutrición, pero tiene que ser por un descuido, porque yo lo digo por mí no se ellas, porque no puedo hablar por las demás, mi niño siempre se me enferma pero nunca me ha caído en desnutrición, ahí está la enfermera del centro que lo sabe, los niños mío nunca han caído en desnutrición ni en bajo peso. Siempre están normales aunque estén enfermitos, porque el cuidado y el tiempo. Si el niño tiene su gripe, el mío porque se pone malito, que ya cuando viene a desarrollar la gripe es porque la tiene estancada en los pulmoncitos y ya tengo que internarlo, pero ya cuando vengo a ver que le está dando la gripecita pues yo, de una vez, lo llevo a tiempo, pues entonces si tú te descuidas…. Si el niño vomita más de tres veces atiende a darle su suerito oral para irlo hidratando, darle su jugo, mucha agua y así no se desnutre, porque mientras va botando líquido, va recuperando ese líquido y no llega a la etapa de desnutrición. Es mi opinión.

**¿Qué piensan los demás?**

-Claro, yo creo que es así, por ejemplo a mi niña una vez le dio vómitos y diarrea, mi deber es hacerle su comidita en la mañanita para comérsela y a las doce, porque tratar de lo que ellos botan, agarra (*los alimentos*) y darle para que se recuperen, porque si uno lo deja, está enfermo y uno lo deja así se va a poner peor y por eso es que hay que alimentarlo, cada vez hay que alimentarlo bien.

**Entonces yo he escuchado que algunos de ustedes mencionaron la basura, y que algunas de ustedes han mencionado que la gripe, problemas al respirar, diarrea, vómitos y amebas y dengue (o enfermedades de los mosquitos) tienen que ver con cosas en el medio ambiente.**

Si.

**Entonces cuando ustedes hablaron conmigo individualmente aquí están algunas de las respuestas que son problemas que ustedes observan en este barrio aquí:**

1. **Basura**
2. **Quema de Basura**
3. **Mosquitos y Agua**
4. **Perros y Animales que están muy cerca de la gente**

**Entonces pensando en estas ideas tal vez podemos discutir un poquito de eso y si ustedes pueden explicar. ¿Cómo se ve la basura, que pasa con la basura en este barrio?**

-Algunos la guardamos en fundas, por ejemplo yo la guardo en funda para cuando pase el camión tenerla en un lugar enganchada para que los perros no las alcancen y así no rompan la funda y así se haga más fácil. Otros se desesperan y no esperan el camión, bajamos aquí, la tiramos, y luego al vecino que le está afectando esa basura ahí agarra y la quema, porque dice que el camión no la va a recoger, lo que agarra y la quema y ese es uno de los problemas.

-El camión casi no viene a recoger la basura. El mismo dura demasiado tiempo para pasar a recoger la basura.

-Hay ocasiones en las que uno se desespera toda esa basura acumulada es lo que llama mosquitos y ciempiés. Uno al tener tanta basura lo que se crían pájaros (insectos) y eso, se pueden entrar a la casa y si el niño está en el suelo, pican al niño cuando están dentro de la casa o a cualquier persona que esté dentro de la casa.

**¿Qué piensan de la quema de basura? ¿Eso pasa con frecuencia o a veces? ¿Cuáles son las consecuencias de esto?**

-Pasa con frecuencia.

-El bajo (*hedor*), entra por la nariz de los niños.

**¿Cómo está relacionado la basura y los problemas de salud en este barrio?**

-Por el problema del asma está relacionado con la quema de la basura porque todo el que sufre del asma pues ahí le ataca la tos de una vez.

**¿Cómo está tener cerca el basurero del barrio?**

-Mal, está muy mal.

-Porque no sé si por allá arriba se siente el humo, pero por donde yo vivo que cuando esa brisa sopla para acá nos trae todo el negro de ese humo porque cuando queman la basura todo ese humo arranca para allá y entonces si uno tiene un niño que tiene asma o se aprieta del pecho inmediatamente inhala ese humo el niño va a empezar a toser y le va a dar problemas para respirar. Entonces, eso nos afecta bastante, entonces cuando la brisa da para acá nos trae todo ese humo.

-Y ese es un problema.

-Serio.

**¿Ustedes piensan que es diferente en otros barrios que no están tan cerca del basurero o es lo mismo en toda Consuelo?**

-Para mí que es aquí nada más, ya que el basurero está ahí mismo y es a nosotros que nos afecta ni a Guachupita que está más cerca llega ese humo, es a nosotros que nos afecta.

**¿Qué piensan ustedes sobre eso?**

-Es así, a nosotros nos afecta más porque estamos más cerca.

-Y no podemos hacer más nada porque, imagínate, no podemos hacer nada por el basurero. No lo van a quitar. Dice que vamos hacer una huelga, porque no lo van a quitar, no lo van a quitar.

- Si uno habla con Johnny él lo quita.

-¿Lo quita? *(dudosa)*

-Sí, yo lo dudo.

-No obstante eso también cuando están quemando la basura el aire se contamina inmediatamente. Entonces, imagínate, los niños principalmente son los más pequeños, son más sensibles y con los poros más abiertos inhalan todo ese humo, toda esa contaminación que botan de la basura, y de ahí ellos lo absorben.

-Y más rápido se enferman los niños.

-Y esos perros de ahí arriba lo tiran ahí y todo ese bajo (hedor) viene.

-Entonces ese es un bajo (*hedor*) que no se aguanta (*de la basura*).

-Mira, tiran perros, es más tiran de todo en ese basurero y toda esa brisa viene para acá.

-Tú sabes, toda esa basura de Consuelo es para ahí que viene, del hospital ahí es que viene.

-Todo, todo, todo.

**¿Qué pasa con la basura en el basurero aquí, o sea, lo dejan aquí y que pasa, se queman o qué?**

-Sí, la queman. (*Todas de acuerdo*)

-Allá mismo ellos le pegan fuego.

-Y entonces todo ese humo nos afecta.

**Entonces hemos hablado de problemas de salud que vemos en este barrio, y algunas cosas en el medio ambiente que tal vez tienen que ver con la salud. Ahora quiero escuchar de ustedes, y esta es la más importante de todo: pensando en el futuro…pensando en estas prioridades de salud y el medio ambiente, vamos hablar como la comunidad puede seguir adelante para abordar estos problemas como los individuos y la comunidad. ¿Qué piensan ustedes: cuales son algunas intervenciones para estos problemas más importantes que nosotros podemos hacer?**

-Yo pienso que ese basurero deben de quitarlo de ahí y buscarle otro puesto, porque a los que más afecta es a nosotros los que tenemos niños.

-Y que el camión de la basura pase semanal.

-Claro.

-Y que todos reunamos la basura y la echemos en una funda para cuando el camión de la basura pase y así están limpias las calles.

-Para mí es todas las mujeres y los hombres que empiecen a limpiar el barrio entero *(risa del grupo),* y que cada quien tenga su ladito limpio y no tiren basura.

**¿Y cómo podemos lograr eso?**

-Oh, colaborando todos.

-Todos juntos. (*Muchas dicen “si”)*

-Hablamos con todos los vecinos un sábado o un domingo y empezamos a limpiar y empezamos a chapear y…. (*risa del grupo*).

-Y otra cosa con esos animales de noche uno no puede dormir con un grupo de animales sueltos. Uno no puede estar en su casa tranquilo ni dormir tranquilo con todas esas vacas, todo ese postrero, y así mismo la empalizada la quieren tumbar.

-Ellos pasan la noche entera suelto y si fuera en un sitio que lo agarraran ellos no lo soltaran.

-Y uno no puede sembrar nada, si uno siembra una mata o una yuca, ellos vienen de noche y se lo comen y uno no puede sembrar nada con eso.

-A veces mi papa tiene que salir a media noche a sacarlo de aquí adentro del patio.

-Y del cesto se rascan, porque no sé qué es lo que le pica si es la espalda (*risa del grupo*).

-Ya lo sabe y entonces.

-Ay Dios mío.

-Oh, pero es verdad, yo oigo el cesto que están como arrancándose como que se tuercen y entonces es en la misma cabecera donde uno duerme.

-Y uno no puede hablar con la gente, porque después se quillan (*enojan*). Se ponen guapo. No, pero uno no sabe cuál es el dueño.

-A veces uno lo ve.

-Si ahora mismo uno no ve ni uno pero ahorita como a las once (*11pm*), para que tu veas que uno no puede sacar ni la cabeza, porque cuando viene a ver te dan un cachazo de la vaca (*risa del grupo*).

**Entonces a lo mejor no es posible de quitarse del basurero o a lo mejor sí eso es posible. Quiero escuchar de ustedes, ¿cuáles esfuerzos comunitarios son los posibles soluciones en este barrio a mejorar el asunto del humo de la basura, por ejemplo?**

-Que lo quiten el basurero de aquí.

-Debemos de concientizar y decir que esto nos está haciendo daño a todos pero la medida la tenemos que tomar entre todos, y entre todos podemos decir: esto nos está afectando y vamos a reciclar, vamos a hacer esto, vamos a hacer lo otro, y así podemos eliminarlo.

**¿Qué piensan ustedes?**

-Claro, es así.

-Si uno no la quema en el barrio, el humo del basurero como quiera nos llega.

-Porque prácticamente el humo de allá es que nos hace daño. Porque cuando llega ese humo, eso es lo que nos hace daño.

-Y hay gente que llevan sus hijos allá chiquitos, allá mismo.

**¿Ustedes piensan que las personas que viven aquí tienen el consentimiento de las relaciones entre el medio ambiente y los efectos en la salud, por ejemplo respiración entre los niños o no?**

-Yo no sé.

-No sé si cuando lo estarán haciendo, piensan en los demás o piensan que tienen que salir de la basura, para mí que no piensan en los demás y dicen: si a mí no me hace daño que me importan los demás.

-Cuando quema la basura, ellos pensarían que lo están haciendo bien o que le están haciendo un favor a alguien.

-Porque no tienen la conciencia, no están concientizados de lo que están haciendo.

**¿Hay alternativas que queman la basura?**

-Sí.

**¿Cuáles son?**

-Ellos cuando la juntan prefieren quemarla porque está muy acumulada, como “me está molestando en el patio y tengo demasiada y el camión no cruza déjame quemarla.”

-Y también hay otro problema por donde yo estoy viviendo: echan la basura así en el suelo ahí atrás donde estoy viviendo, lo tiran.

**¿Hay algunas personas que hacen reciclaje?**

-No se hace.

-En el mismo basurero ellos recogen los potes (*botellas plásticas*).

**¿Ah, y todo va al mismo lugar?**

-Sí, ellos lo tiran todo junto.

-Pero aquí no hacen eso (*reciclaje*).

-La gente lo recogen allá.

-Sí, allá. Pero uno en la casa no lo hace.

-En la casa no, uno no lo hace.

**Lo recogen allá, ¿y que hacen?**

-Lo venden.

-Pero eso es allá en el basurero, pero aquí, aquí no lo hacemos.

**Eso sería posible de hacer en las casas, si las personas aprenden hacerlo, ¿Qué ustedes creen?**

-Y las latas ellos también la recogen.

**Si, a veces se pueden vender o llevar a un sitio, ¿eso funcionaría aquí en este barrio? ¿Piensan que las personas estarían interesadas en hacer eso o no importa?**

-Ellos lo cogen allá, y ahí mismo lo juntaba, el camión viene, y lo compra y se lo lleva a otra parte.

-¿Aquí en este barrio?

**Sí.**

-Claro a mí me interesa hacer eso, a mí me interesaba hacer cositas, con esos mismos potes, uno lo rompe y hacer muchas cositas bonitas, me gustaría.

**¿Y qué hacen con esas cositas que hacen?**

-Lo ponen en sus casas un ejemplo: hacen flores con un pote (*botella plástica*). ¿Tú te acuerdas del que tú compraste?

-Sí, yo compre (*risa del grupo*).

-Lo ponen en la mesa, en un florero y a veces lo ponen encima y se hacen muchas cosas, en el estante, ¿usted lo ha comprado eso?

**¿Hay algunas personas que hacen eso en el barrio ya?**

-No.

-Diría yo: que tomando la medida y viendo que eso si tú lo tiras te puede hace daño pero si tú haces algo con ella ya no va afectar, diría que yo que ya las personas tienen mucho más entendimiento.

**Porque a veces una persona hace reciclaje o hace algo con la botella o cualquier cosa, por lo menos hay menos cantidad al quemar, si van a quemar como quiera. Si hay algunas cosas menos en la basura, y si se hace algo con esas partes de la basura, hay menos que quemar.**

-Yo vi en la televisión que hicieron una cartera con esta funda de los jugos de sobre, mira--eso estaba más lindo, con esos jugos de diez pesos, agarran muchos de eso y hacen unas carteras más chulas con eso que hicieron. Pero esas son gente que saben.

-Si ellos le enseñan a uno, uno aprende y así ocupando su tiempo en algo productivo.

**¿Ustedes piensan que sería de interés si una persona pudiera venir a enseñar y crear un grupo de mujeres y hombres para hacer esas cosas?**

-Hay sí, claro.

-Sería bueno.

**Antes ustedes dijeron que pueden buscar un día para que todos limpien su propio lugar y ustedes estaban riéndose mucho de eso. ¿Creen que eso sería posible o no posible?**

-Posible.

-Nosotros lo hacíamos.

-Nosotros lo hacíamos en antes no sé qué está pasando que ya no se está haciendo.

-Al principio uno lo hacía.

-Por lo menos duremos un mes haciéndolo y cada quince días que venía un grupo de hombres y mujeres y limpiaban los potes y las matas, la podaban y estaban muy bonitas, pero todo eso se descuidó.

-Yo no sé lo que paso.

-Pero eso se descuidó y está en el abandono totalmente.

**¿Entonces eso paso en el pasado?**

-Sí.

**¿Ustedes piensan que las personas aquí tienen el poder de hacer un cambio? ¿Cuál es el pensamiento, no sé si me explico, si hay el sentimiento en que algún individuo de hacer un cambio?**

-A mí me gustaría que hicieran algo productivo para uno y así uno puede desarrollarse y así ocupar su tiempo en algo, en el barrio como un taller de algo para uno aprender.

**¿Y cómo podemos lograr eso, que necesitamos hacer?**

-Oh! Unirnos, la unión en grupos y hacerlo y ponernos para eso y así todos con la mente positiva.

-Y lo que dijo.

-No porque nos basamos a lo que una persona hacía, pero no le dimos seguimiento porque esa persona nos encaminó y nos dio la facilidad y nos enseñó que haciéndolo así se hacía bien. Entonces como esa persona se retiró ya todo el mundo se retiró y no debió de ser así, debimos seguir.

-Para adelante.

-Se cansó y como no vio.

-El ánimo, entonces decidió dejar eso y nosotros lo que teníamos que hacer era seguir y no lo hicimos.

-Ya se le quito el ánimo a la gente.

**Si hay un grupo de personas que tienen más conocimiento o un poquito más de entendimiento de cuidar o limpiar, el cuidado de la basura y enseñar a lo demás, ¿Eso sería posible o de interés?**

-Sí, eso sería interesante, porque a veces las personas hacen las cosas y no saben lo que están haciendo y después que lo demuestran y ven el daño que eso hace, entonces ahí toman medidas.

**¿Tienen otras ideas de cómo mejorar estos problemas de salud y del medio ambiente, otras ideas para la comunidad, un individuos, vecinos, para pensar en el futuro de nuestros niños? Quiero escuchar si hay más ideas o pensamientos.**

-Yo misma deseo terminar mis estudios para en el mañana trabajar y así sacar a mis hijos hacia adelante. Es bueno cuando una madre lucha por sus hijos que usted vea que usted le va a sacar provecho a sus hijos, si lucha por ellos. Es bueno cuando usted lucha por sus hijos ver ese anhelo, ese deseo y cuando su hijo se levanta y ve ese deseo de lo que usted hizo por ellos para el mañana.

**Y hablando de niños, ¿en las escuelas enseñan sobre el medio ambiente o como cuidarlo?**

-Sí, hablan de eso.

**Ustedes han dicho que hay muchas personas que no conocen mucho de los efectos.**

-Porque la mayoría, o sea casi, ¿cómo diría?, que el cincuenta por ciento de las personas que viven aquí, todos no fueron a la escuela. Muchos han aprendido algo ya después que han cogido clases así y usted sabe que no es lo mismo. Si no van a una escuela, más o menos lo que le enseñan son letras de cómo escribir su nombre y así se quedan ahí, pero cuando van a escuela y ahí cogen todas sus materias, sí hablamos de naturales. Ya saben que estamos hablando de medio ambiente y cuando cogen sociales ya saben que estamos hablando sobre la sociedad y entonces por eso fue que dije que hay muchas personas que no tienen casi conciencia.

**Y si hay muchas que no han asistido a la escuela, ¿cómo pueden concientizar ellos en el barrio? ¿Cuál sería la manera?**

-Se pueden apuntar en una escuela de noche.

**O sea, para compartir conocimientos en el barrio, ¿hay una manera de hablar con vecinos?**

-Sí, se les puede hacer una reunión y así ellos se reúnen y se les habla, o sea, a los que les interesa, porque hay muchos que siempre están abnegados y siempre es así, una parte que nunca quieren aprender nada y así, pero hay otros que aunque estén mayor si quieren y tienen ese anhelo de si yo quiero aprender, yo quiero ir a esa reunión, yo quiero saber de qué se trata y es así.

- Hay vecinos que se ponen a quemar basura y cuando coges para allá tienes que ir preparados con los puños, no porque tu vayas a decirle: la basura no se quema, tu sabes como es y uno no puede.

-Y eso es un problema.

-Claro, eso es verdad.

-Lo que iba a decir en cuanto a la enseñanza que muchos dicen: que mi tiempo de estudiar ya pasó, sino lo hice en el tiempo que podía pues para que ahora que ya no tengo tiempo para hacerlo.

-Bueno, manos a la obra! Entonces “las mujeres con su escoba y el hombre con su machete!” (*risa del grupo),*

-Claro y a limpiar el barrio para que hayan menos enfermedades.

-Necesitamos el barrio limpio, por los niños y por nosotros mismos.

-Así mismo.

**A veces cuando hay un grupo de personas con motivación puede dar ánimo a los demás, hay esfuerzos en números o en grupos…**

-Así siguen creciendo y así se van uniendo y cuando vienen otros y ven lo que estamos haciendo se unen.

-Lo que pasa es que cuando yo vivía en el batey Alejandro Bas, cada quince días nosotros limpiábamos. Pero no es lo mismo aquí, porque cada quince días nos cobra a nosotros (*risa*). Catalina nos da una a cada una y entonces uno limpia allá está bien. Pero tu limpias aquí y no te dan nada y así uno no quiere limpiar.

**¿Cómo podemos motivar a una persona para hacer el esfuerzo, para hacer eso, hacer el esfuerzo que no sea ofreciendo dinero, hay otras maneras de motivar?**

-Claro.

-Sí.

-Hay otras maneras.

-Como hacen aquí.

-Sí, nosotros limpiábamos antes.

-Bueno nosotros limpiamos bien sí. (*Risa*).

-Sí.

-No es mentira, sí.

-Es verdad.

**¿Y por qué no funcionaría aquí o si funcionaria aquí?**

-Aquí duramos como un mes limpiando.

-Yo limpiaba.

-Yo también y pasaba con mi escoba el día entero barriendo.

-A mí no me importaba y yo limpiaba en mi casa y en todos lados.

-Y yo también.

-Solo es cuestión de imponerse.

-Hay que hacerlo. Hay que hablar con Hilario. Él está dispuesto. Ese hombre está dispuesto a eso.

**¿Hilario es?**

-El presidente (*de la junta de vecinos*). Uno va y le dice: vamos hacer esto, vamos hacer aquello, ese hombre siempre está disponible para todos.

-Porque lo que a él le gusta es que la gente tenga motivación y que no lo dejen solo.

**Y cuando un grupo de personas va donde Hilario, ¿las cosas cambian?**

-Sí. El quiere.

-Porque ese hombre siempre está dispuesto y si es para la comunidad. Siempre está dispuesto.

-Cada quince días *(con limpieza*). Se sacaba un domingo: los hombres a chapear los palos y quitar todo eso, pero hay mucha gente enredados.

-Él nos daba motivación.

-Y él hasta líos, se encontraban mucha gente que le mochaban sus palos.

-Que era para la empaliza los palos y se enojaban.

-Porque la limpieza es para todos.

-Lo que hay que hacer es hablar con la gente. ¿Tu estas dispuesto a limpiar su palo? Si, y pues vamos a limpiarlo. Y tu no? Pues vamos a dejarlo así. Y peguntárselo a cada quien y a su patio usted quiere? No, ok está bien vamos para otro lado.

-Es que uno no puede dejarlo ahí, porque va afecta a uno. Entonces y por eso uno no puede dejarlo ahí, ¿No verdad? Si uno va a limpiar hay que limpiar todo, o no.

**Entonces, ustedes han hablado de muchas ideas y cuáles de estas ideas son más probables de hacer un cambio para la mejoría?**

-Hablar con Hilario.

-Limpiar el barrio y que nos quiten el basurero, eso es lo que queremos.

-Hay sí, el basurero.

-Cada quince días—quince persones con machetes (*y limpieza*).

-Y así se va ese mal olor.

-Recoger la basura y echarla en fundas.

**Ya ese es un asunto de conocimiento y de cómo aprender.**

-También queremos aprender.

-También necesitamos eso.

-Aprender hacer flores.

-Que en vez de uno estar el día entero en una silla haciendo cuento (*nada que hacer*), y así ocupar su tiempo en algo, claro en algo productivo.

**¿Hay personas que están en casa todo el día y les gustaría tener algo que hacer?**

-Si (*el grupo de acuerdo*)

-Sí, porque después que hace los quehaceres domésticos, lo que uno se pone es hablar y ver el televisor, porque después que uno hace su oficio es hacer cuento que uno va, pero si uno hace sus oficios y dice a tal hora vamos para el curso tal, y así ya tenemos en que ocupar nuestro tiempo en algo productivo.

-Uno aprende, porque en el mañana uno no sabe si va a vivir de eso.

-Claro y de eso yo puedo vender y hacer cuarto (*dinero*), y ganarse unos chelitos.

-Es verdad sí.

-Claro.

-Una mujer vendió, ¿en cuánto fue la flor que te la vendió?

-Cien pesos.

-Bonita.

-Por allá hay una mujer que hace flores con potes.

-Esa misma, de allá atrás.

-Sí.

-Claro.

-Todos los colores.

-Pero nosotras aquí, no tenemos nada en que ocupar nuestro tiempo. No hay nada.

**Entonces, ¿hay algo más que ustedes piensen que es importante y que tal vez no hemos discutido sobre la salud de los niños y de las familias en este barrio y la relación con el medio ambiente?**

-Come bien, bebe jugo. Claro, y es bueno.

-Tener los niños bien limpios, porque hay niños que son las diez de la noche y están sucios, en la calle, con los pies, la ropa sucia. La madre atender a sus hijos.

-Y darle menos cosas dulce y más comida con sal, ya que eso trae muchos parásitos, los niños que están así con esa barriga grande es solo de comer cositas así y disparaticos, no es verdad.

-Y el culito flaco.

-Y el culito chupaito y todo se le va para la adelante y todo eso es parasito, porque uno le da una galleta y un chin de jugo y ya acabo, y es lo que le da muchos parásitos.

**Bueno, pues si no hay más cosas que decir, voy a decir muchas gracias por su participación, y muchísimas gracias por venir.**

-Gracias a usted por su tiempo.

-Bueno, la conversación es en beneficio de nosotros mismos.
