# Supplementary material for: Talking trash: Perspectives on community environmental health in the Dominican Republic
Source: PLoS One. 2021 Mar 29;16(3):e0248843. doi: 10.1371/journal.pone.0248843 (PMC8007031; doi:10.1371/journal.pone.0248843)
Supplement: S4 File — (DOCX) [file pone.0248843.s004.docx]

**(*Introducción*)…**

**…Ustedes compartieron sus opiniones sobre problemas de salud más comunes… (*Explicación*)**

1. **La gripe**
2. **La fiebre**
3. **Diarrea/Vómitos/Amebas**
4. **Dengue**
5. **Erupciones/Infecciones de la piel**

**Vamos a discutir en grupo.**

**¿Qué piensan ustedes de esta lista cuales son las más comunes que están en este barrio?**

-Gripe, enfermedades de la piel y diarrea.

**¿Por qué?**

-Gripe porque hay muchas bacterias y muchos virus en el aire y los muchachos agarran muchas cosas con las manos y se la entran en la boca y no tienen que ver de donde sea. La diarrea es por algún alimento que comen que le hace daño y vomitan. Las enfermedades de la piel, algunos la tienen desde nacimiento y así diferentes.

**Gracias y ¿qué piensan los demás, algunos aquí son más severos que otro?**

-Bueno yo estoy de acuerdo con lo que ella dice eso es lo que hay más aquí.

**¿Cuál es lo más común que ustedes ven o unos de esos que tenemos que mencionar?**

-Mosquitos.

**¿Mosquitos?**

-Claro está muy abundante ahora mismo.

-Lo más común de eso es la gripe porque usted sabe de ahí hay la gripe le da a uno, uno que grande y chiquito esa es la enfermedad que más fácil le da a los muchachos y que las personas grande puedan tener.

**Es diferente, ósea, ¿hay más tipos de estos virus que hay aquí en comparación a otros barrios o en todo el mundo hay mucha gripe, que piensan?**

-Es como dice ella hay personas que pasan por una mata y por eso le da alergia o si le cae un poquito de agua lluvia también le provoca la gripe. La gripe con cualquier cosa usted se puede cuidar y curar de la gripe porque eso es lo más fácil que uno puede tener.

**Usted dice que a lo mejor es lo más común ¿hay otro que sea más común?**

-Fue la chinkungunya. Creo que acabo con el que la tenía.

-Esta esa que paraliza el cuerpo de los muchachos también.

-También está las amebas a veces no hay agua buena y ellos toman agua mala y también se enferman por esa de la ameba.

**Y los que son más severos, ósea algunos son más comunes le dan a muchas personas pero otros son severos que pueden agarrarte y llevarte al hospital ¿cuáles de estos son?**

-Diarrea y vómito y ameba. Y el dengue.

**Entonces vamos a pensar en estos y ustedes también compartieron sus opiniones sobre el medio ambiente del barrio, y como el agua , el aire , las casas, letrinas, las matas, todo está alrededor de nosotros y como nos afecta y como afecta la salud de los niños. Aquí tengo un listado de los problemas más comunes que ustedes mencionaron y sabemos que muchas veces lo que está en el medio ambiente afecta a la salud de los niños. Entonces ustedes mencionaron estos que son problemas del medio ambiente que hay aquí en nuestro barrio:**

1. **Pozos de aguas**
2. **Problemas con letrinas**
3. **La basura o acumulación de la misma**
4. **La quema de la basura**
5. **Los mosquitos**
6. **Los perros**

**Quiero escuchar de ustedes como en el grupo ahora que piensan de este listado.**

-Bueno, hay muchas gente que tienen letrina y todo lo tiran por ahí. Por donde yo vivo, hay una zanja y todo pasa por ahí y siempre dicen que lo van a limpiar pero esa agua enferma los muchachos. Muchos perros que andan por ahí llenos de nacidos.

**¿Cuáles son las experiencias de los demás?**

-Los mismos perros que están llenos de rasquiñas y eso da alergia. Esos animales enferman el aire y el ambiente cualquier carajito se le pega. Ya se le puede pegar esa enfermedad del perro entonces.

**¿Hay más perros en este lugar?**

-Si hay muchos perros enfermos y rasquiñosos. Eso contamina el aire. La misma basura acumulada también trae los mosquitos, pila de enfermedades.

-Los pozos de agua, ósea las zanjas de agua negras. Hay mala administración del agua porque a veces ponen un cubo debajo de la llave y eso dura horas botando agua, se llena un tanque, y eso sigue botando y sigue botando. La falta de letrina--hay personas que queman la basura, otros la echan en fundas y la sacan los martes cuando viene el camión. Entonces, cuando se acumulan basura ahi se producen mosquitos porque ponen basuras y se llenan de agua. Ahí se va produciendo el mosquito y los perros que hay gente que creen que son dioses y quieren tenerlo en todas partes.

-Yo vivía donde hay perros, y hay demasiados perros en el barrio pelados. En el patio donde yo vivo, hay más de diez niños y hay perros allí adentro pelado y se le pegan perros de allí cogen para adentro de la casa de uno eso trae mucha enfermedad. Yo misma tengo una enfermedad y eso yo sé que es mal y que hay que hacer algo con respeto a esos perros. Lo de los pozos del agua, hay un pozo de agua donde mi madre, eso es en la puerta que se para, eso es una agua verdecita que yo diría que eso hay muchos mosquitos y a un sobrino mío lo picó un mosquito, él tenía dengue y yo digo que eso viene de esa misma agua.

-Para mí la quema de basura también afecta mucho el medio ambiente y a las personas y a los niños que se aprietan le hace mucho daño. La acumulación de basura también trae muchas enfermedades y los pozos de aguas negras traen muchos mosquitos.

**Entonces ustedes mencionaron dengue y el chinkungunya también.**

-Sí, algunas enfermedades de mosquitos. Ahora mismo hay mosquitos en todos lados.

-Tú puedes tener mosquitero y como quiera traspasan.

-Y donde hay mucha hierba también.

**¿Qué pasa con la basura aquí, ósea, algunos mencionaron lo que hacen algunas personas, pero cuales son las cosas que hacen la basura las otras?**

-El camión para la basura pasa los martes pero a veces hay personas que no tenemos tiempo para botarla y la tiramos en todo los lados y los muchachos comienzan a jugar y los perros la sueltan y a veces tienen pampers y eso es algo que tiene mucho microbio y nos hace daño a todos.

**Entonces viene el camión…**

-El camión viene los martes.

-A veces viene hasta el miércoles en la tarde pero hay veces que el viernes todavía siguen cargando basura.

-Ellos no les importa que tiene adentro, cuando llega la noche los perros comienzan a arrastrarlas y eso es una contaminación para el barrio, porque cuando uno tiene que pasar por donde hay basura y no es de uno perro a ellos no les importa, el fin es que ellos la sacan de su patio y se la ponen al tormento de otro. Hoy pasó, pero cuando tú pasas como quiera vas a encontrar un saco, aunque no sea día de botarla, ellos no son conscientes.

-Algunas personas no son conscientes.

-Y ahí atrás a donde yo vivo yo creo que esa gente no sacan basura porque a todas horas tú ves el basurero a veces son las once y tú ves toda esa basura y ese mal olor que nadie lo aguanta.

-Y por mi casa en el mismo barrio en el pedacito de donde mi mama, vive más de cincuenta gente y si hay cuatro baños en total. Hay muchos por ahí y no hay baños.

-Imagínate, (*va al baño*) donde quiera tu haya una funda y la tiran.

-Imagínate cuando amanece.

-En los sacos con la basura.

**Entonces, cuéntame donde están las letrinas, o donde van al baño las personas que no lo tienen.**

-Bueno, por donde yo vivo, hay personas que van, y no van diario, esas persona van menos me entiendes, y a veces esperan la noche y a veces hacerlo en funda también.

-A veces aparecen fundas hasta arriba de las casa.

-Sí y por allá.

-A veces no.

-Si aparecen uno no puede ni coger agua lluvia.

-El que va de noche donde cayó. Pero si quita el problema de uno, se lo pone a otro. Un día había un olor, y yo busco y busco y busco en casa, en la cortina y la lavo y echo mistolin y cloro y sigo buscando y buscando pero alguien me dice sube un muchachito arriba del techo.

-Y allí estaba.

-Sí, allí estaba ese “bizcocho” (materia fecal).

**Entonces hay algunas letrinas…**

-Si hay, pero hay muy pocas para la cantidad de persona que viven en ese círculo. Hay muy pocas porque hay casas de esas que tienen cinco y seis personas y otras que tienen cuatro y otras que tienen tres, y son muy pocas la que tienen una sola persona. Entonces un sola baño… Y vamos a ver en el estado que este el baño porque eso es otra cosa que afecta al medio ambiente.

-Porque yo conozco un caso de un baño que ya la gente no quieren ir porque hay un hoyo y la gente dicen que cuando viene a ver se van por el hoyo.

**Peligroso. ¿Y porque hay tantos pozos de agua?**

-Cuando llueve y el agua no tiene salidas, y no tiene por donde salir, el agua se queda aposada y ahí ya usted sabe.

-Hay que darle hasta dos semanas de sol para que se pueda secar de aquí a dos semana ya usted sabe cómo se pone eso.

-Y a veces abren una llave y comienza a botar agua y no tiene por donde salir y se atasca y ahí se crean los parásitos.

**¿Hay otros problemas que haya en esta comunidad que no hayamos mencionado? ¿Ustedes piensan que estos son los más importantes o hay más que debemos hablar?**

-Bueno para mí es la basura, el agua sucia, y los mosquitos, eso es lo peor que hay por aquí.

**Entonces gracias por lo que ya han compartido y ahora pensando en estas prioridades de la salud y del medio ambiente, vamos a discutir como personas que son miembros de esta comunidad, ¿qué podemos hacer para poder seguir adelante, y abordar estos problemas en la comunidad? Quiero preguntarles: cuáles son algunas intervenciones que ustedes piensen para esos problemas más importantes?, ¿Cosas que la comunidad puede hacer para mejorar los problemas un poquito?**

-Bueno, primeramente los baños.

-Y los perros, demasiados perros hay.

-Y así los baños: las personas no lo atienden. Y todo esos perros callejeros que hay uno podría recogerlo y mandarlo a botar, no se, o a trancar. Eso lo podríamos hacer y eso sería lo mejor.

-Bueno habrá que comenzar.

-Los mosquitos no lo digo porque esos no los mata nadie. (*risa del grupo*)

-No, es una realidad: tu fumigas ahora mismo, y al rato vienen como en manada.

-Se van por rato.

-Contra eso no hay cura.

-Con la limpieza, se puede eliminar eso. Concientizando el dueño de un patio y de un baño, concientizándolo en que hay muchas gentes y tiene que a ver más de un baño y que tiene que tenerlo en buen estado.

-Concientizando a la gente, diciéndole que si el camión pasa, no tienen por qué estar sacando basura y que si pueden tener su saco amarrado porque hasta la persona que recogen la basura se quejan y no quieren venir a recoger la basura. Por uno pagamos todos porque echan de todo de lo que no tiene que echar en la basura entonces eso habla mal del barrio. Las personas que tienen perro, imagínate, que lo tengan amarrado porque una sola perra pare hasta ocho, siete, y seis perritos ya esos perros eso es fuerte. Entonces de esos siete, hay tres hembras, y empiezan a parir otra vez uno esta full de perros.

-Para mí, lo que más molesta son los pozos de agua y la cuestión de los baños y de los sanitarios. Para mi es eso.

**Entonces si usted pudiera cambiaría algo, ¿Que haría?**

-Los pozos de agua.

**¿Qué haría para cambiar eso?**

-Si yo pudiera desalojara a todo el mundo, si Dios me ayudara, porque imagínese, no se puede hacer otra cosa.

-Imagínese usted, yo sería la primera.

**¿Cómo podemos trabajar juntos la comunidad para mejorar uno de estos problemas? Osea habían mencionado que ponerlos perros afuera o concientizar la personas…**

-Bueno, eso se da uniéndonos todos pero lo que pasa es que a veces, ella quiere, la otra quiere, y ella también quiere, pero así como ellas están de acuerdo, hay otros que no lo están, y eso es lo que pasa en realidad.

**Entonces hay personas que no están de acuerdo con algunas cosas.**

-No, porque si yo echo una cosa ahí y esta muchacha me dice no, no eche eso ahi que eso me molesta, yo no lo voy a hacer. Pero si le dice a otro mañana y pasado vuelve hacerlo otra vez y eso es lo que pasa.

-Yo he estado barriendo en mi frente y la gente me ven barriendo y pasan con una funda y la siguen tirando. O sea, como que el tiempo que yo dure en barrer a ellos no le importa. En mi frente, llega fundas de Cheetos, Quesitos, y yo no tengo colmado, pero es que la gente lo va tirando aun tú le ponga un zafacón o vean un saco. Porque mira, hay muchos que tienen conciencia y hay otros que no tienen conciencia. Hay gente que se ofenden si tú le dice, “no me eche esa basura ahí, eso es un camino y es libre,” pero yo cojo mi escoba y lo barro.

**Entonces, ¿cómo pueden motivar o dar ánimos a los demás, para que no echen la basura por aquí? Porque sabemos que es difícil decir esto a nuestros vecinos a veces…**

-Ofendido.

**Pero hay una manera para hablar con los demás de eso? O hay un grupo que puede ayudar?**

-Eso se puede hacer haciendo una reunión o la junta de vecinos.

-Exactamente.

-Una reunión para ponernos de acuerdo todos para saber lo que se vaya hacer.

-Y poner contenedores y ahí echan de todo.

-Si una vez lo pusieron y encontraron basura hay de todo y lo quitaron otra vez.

-Si, ponen un tanque para que la gente que va pasando eche la basura. Aquí se pusieron tanques en varios puntos se pusieron tanques una vez, y se hicieron con hoyo para que no se llevaran los tanques porque si lo dejan bueno se lo llevan. Nadie quería el tanque en su área pero el camión pasaba hoy y ya en la noche el tanque estaba lleno. Entonces no era nadie y quitaron los tanques. Era para el que va pasando vaya echando la basura.

-Para que no hubiera tanta basura regada pero es que pero nadie lo quiere en su puerta en la esquina del colmado. No lo quieren porque ya saben lo que viene en la noche y en el otro sitio no lo quieren. Así nadie lo quiere porque el tanque al otro día amanece lleno y nadie sabe quién fue que lo lleno.

**Entonces con más tanques sería mejor ¿o no?**

-Bueno, concientizando a las personas.

**Aja, y ¿cómo podemos concientizar las personas?**

-Diciéndole lo que afecta el problema que si tienen un paquete de potes regados que eso acumula agua y esa agua puede provocar dengue. Y que no acumulen tanta basura, que la echen en funda o en saco y que la mantengan amarrada, para no seguir contaminado el medio ambiente. Que mantengan su frente limpio y su área limpia. Que cada quien mantenga su área limpia.

**¿Qué piensan los demás?**

-Para mí lo más importante es que la personas limpien los baños y que vayan arreglando esa tuberías que estén rotas por ahí y tapen esos hoyo que hallan, donde se acumula el agua. Bueno, y después de eso puede comenzar lo otro.

**Y si queremos más baños para el barrio, ¿cómo podemos hacerlo o con quien podemos hablar o cual es el proceso o hay un proceso**?

-Hay un proceso. Lo tiene la junta de vecinos y las personas tienen que ir.

-Para hablar con el Síndico.

**Y ustedes creen que eso proceso funciona o no?**

-Todo depende como se haga.

-Pero hay que intentarlo.

-Hay personas que tienen donde hacer el baño pero no tienen la posibilidad. Mi mama tenía una casa pero no tenía baño, y ella saco un pedacito e hizo el baño. Hay otro que lo quieren hacer pero no tienen con qué. No tiene la posibilidad, me entiende y eso es. Donde yo vivo, vivimos doce personas y hay un solo baño y pudieran hacerse porque hay un patio y pueden hacerse tres y cuatro baños porque somos demasiadas personas pero es la posibilidad. En el patio donde está mi nieto, viven más de diez personas y hay un baño que el hoyo se está casi derrumbando para abajo y no hay posibilidad, pero todo el mundo quiere vivir bien, nadie quiere vivir mal.

**Entonces un grupo ha ido a la junta de vecinos hablando de eso?**

-No.

-No.

**No, es solo por curiosidad.**

-No se ha ido a la junta de vecinos en cuestión a esos baños porque mayormente son casas de alquileres que tienen un dueño. Se supone que el dueño del patio es que tiene que acomodar al inquilino. Entonces eso ya no está en la mano de la junta de vecinos porque yo que tenga doce casas de alquiler o quise casas y me pagan mi dinero yo no puedo ir a la junta de vecinos para que me faciliten un material para yo poner un baño, porque ellos están pagando y se supone que el dinero es para mejorar.

**Ok, si están en una casa alquilada, es diferente. Entonces, ¿Cómo vecinos que viven ahí juntos, que puede hacer un individuo para hacer un impacto en el barrio? ¿Creen que un individuo pueda hacer algo, o no, o tiene poder de cambiar algo o que opinan ustedes?**

-Como una persona que pague alquiler que si puede.

**Si cualquier persona que viva en este barrio, cualquier persona puede hacer un cambio o no es posible hacerlo, si es una persona o necesita un grupo, no sé cómo decirlo.**

-Eso es mejor que lo haga un grupo porque un solo no podrá hacerlo.

-Aquí dicen siempre eso y nunca lo hacen.

-Hacer campaña, digo yo.

-Si los políticos dicen arreglaremos aquí arreglaremos ya.

-Y cuando llegan a donde quieren se olvidan del barrio.

**Entonces ustedes mencionaron que si cada persona mantenga su área limpia puede tener su barrio completamente limpia y ¿por qué no pasa eso? ¿Que necesita cambiar para cada persona tiene esta conciencia?**

-Lo que hay que hacer una unión con todos. Pero si no hay unión, no hay nada. Pero si hubiese unión un vecino con otro que si ven una funda digan vamos a recogerla pero no a mí me molesta y le molesta al otro pero si el otro ve una basura lo que tira es otra basura para estar acumulada pero es que le gusta la suciedad. Y eso lo que trae es enfermedad y eso es lo que pasa. Quizás otro del otro lado quizás la recoja porque él quiere su barrio limpio, pero el que es natal no lo hace mejor ensucia más ante de recogerla.

**Hay personas que reciclan cosas como: botellas de vidrio o reciclan y llevan a un sitio, ¿o hacen otra cosa?**

-Yo la recojo y la funda también.

**¿Ustedes saben lo que es abono o abonar desechos de frutas o vegetales o algo que puedan echar en la tierra y con un proceso de mezclar poco a poco se convierte en tierra otra vez? ¿hay persona que lo hacen aquí o no?**

-No. No.

**No, es solo por curiosidad porque pensando en todas las basuras que han mencionado.**

-Aquí lo que usamos es muchos potes para venderlo y se lo llevan para la capital.

**Ok, se lo llevan para otra ciudad.**

-Exactamente. También se llevan las sillas rotas, hoja de lata, a veces los muchachos la recogen.

**Y se puede vender?**

Si.

**Entonces en este barrio cuales esfuerzos comunitarios parecerán pasibles hacer? Hemos hablado de que tenemos muchos problemas, y a lo mejor, falta de conocimiento o falta de motivación o problemas con el espacio, problemas con cualquier cosa. ¿Cuáles son las fuentes comunitarias que a lo mejor funcionarían?**

-Bueno, eliminar todos esos perros. Y los mismos baños también.

-Y también ir a las casas de esa gente que viven en alquiler y los dueños. Yo digo que deberían estar presentes para que escuchen porque eso le pertenece a ellos y ellos pueden pedir una ayuda. Así terminan lo que ellos a veces comienzan, o sea, porque es necesario tener una casa que tenga un baño.

**Sí, es un derecho.**

-Sí, es un derecho y un deber que las personas deben de tener.

-Las personas que viven aquí deben exigirle a los dueños que eso le hace daño, porque si ellos no le exigen a ellos que son dueños, entonces las personan que alquila necesitan un baño y ellos harán la diligencia de hacerlo. Pero si no se le ve el interés y ellos ven que llega el mes y pagan y no piden, no dicen nada y el dueño se queda tranquilo.

-Entonces yo digo que los dueños que tiene casa en alquiler ellos deberían venir y escuchar para ellos saber que es necesario.

**Entonces lo mejor sería compartir algunas ideas con los dueños que realmente tienen poder de hacer un cambio? Y con los mosquitos, ¿que podemos hacer como individuos?**

-Mire, en tiempo de agua, ellos se reproducen más, usted me entiende, porque se aposa el agua. Eso es como yo le dije eso quien lo elimina nadie para eliminar eso. Hay que eliminar los posos. Vamos a suponer que llueva y que ya al otro día este seco, pero si el agua se acumula y dura dos y tres semanas hay ya viene un grupo de parásitos como los mosquitos y dengue de toda esa agua.

-Donde hay muchas matas, también le gusta a ver muchos mosquitos. Donde hay muchas hierbas. La basura también trae muchos mosquitos.

-Se reproducen con toda la basura.

**Algo más?**

-Yo creo que no.

**Bueno…gracias…etc…**
