# Supplementary material for: Talking trash: Perspectives on community environmental health in the Dominican Republic
Source: PLoS One. 2021 Mar 29;16(3):e0248843. doi: 10.1371/journal.pone.0248843 (PMC8007031; doi:10.1371/journal.pone.0248843)
Supplement: S5 File — (DOCX) [file pone.0248843.s005.docx]

***(Introducción)…***

**…Ustedes compartieron sus opiniones sobre problemas de salud más comunes… (*Explicación*)**

1. **La gripe**
2. **Parásitos**
3. **Diarrea/Vómitos**
4. **Enfermedades de la piel**
5. **Asma**

**Vamos a discutir en grupo.**

**¿Qué piensan de estos problemas--son problemas aquí en [el barrio]? Hay más problemas que no tenemos aquí en el listado (gripe, parasitos, diarrea, vómitos, enfermedades de la piel y asma)?**

-Pero hable uno ohoh.

**Entonces…si…**

-Ahora mismo lo que más se está viendo aquí en [el barrio], por las lluvias y todo eso, es la gripe, bueno yo digo en mi casa.

**Y eso es porque hay mucha lluvia? Que piensan los demás? Hay más cosas que afectan la salud de los niños o algunas razones para llevar a los niños al médico que son muy comunes aquí en el barrio?**

-La niña mía tiene una enfermedad en la piel y la lleve hoy porque le está dando fiebre.

**Como algo en su piel, ok. ¿Eso es común aquí?**

-Yo no sé. La mía lo tiene, yo no sé los demás.

-Lo que afecta más en este barrio es especialmente la limpieza y eso a través de los mosquitos porque la gripe prácticamente es una cosa natural porque cuando da gripe le da hasta a uno, pero la forma de cómo está esta comunidad, ahora mismo con toda esta lluvia y en todo los lados lodo y los mosquitos es por todos los solares que están sucios y por la hierba y pueden permitir cualquier enfermedad.

**¿Y qué piensan los demás? Cuales son algunas enfermedades más severas que a lo mejor afectan menos niños pero son más severos con los niños que la tienen?**

-Yo digo que...

**Y no tenemos que hablar de lo que está aquí, si piensen en otra cosas. Eso fue solo para empezar.**

-Si pero viendo ya lo que tienen aquí, el asma es un poquito más complicado.

**Ok.**

-Aunque no se anda viendo mucho aquí pero eso ha traído problemas y muchas veces también viene de nosotros porque no nos cuidamos con algunas cosas.

**¿Cuáles son algunas cosas?**

-El que es asmático, el polvo le hace daño pero también el humo y muchos aquí en la comunidad no acumulamos la basura para el camión--muchas veces la quemamos y ese humo nos hace daño y la mayoría de aquí de los vecinos fuman, y eso le hace daño a uno.

**¿Y que piensan ustedes o cuales son las experiencias de los demás? No tiene que estar en este listado, no tengan vergüenza aquí, eso es para ensenarme. Ustedes son los expertos…**

-Yo creo también que son los vómitos y la diarrea. Tú sabes que los niños andan demasiado descalzos y ahora mismo esta este bache y esta lluvia, tu sabes que hay muchos parásitos y eso afecta mucho a los niños.

**Ok. Alguien más?**

-Bueno yo tenía diarrea y no me hizo nada y ayer pase el día entero.

**Ok. Entonces eso es un problema que está bastante?**

-No sé por qué nadie más tiene y uno es mayor. Uno es que tiene que controlar la gripe porque si no, la gripe va a matar a uno. Eso a veces me pone con los ojo haya atrás y me pongo a comer.

-Y le gusta mucho eso.

-El que comer si a veces estoy full y voto el resto porque estoy lleno.

-Ósea que si tu relajas compra una libra de arroz no da para ti y el.

-No tanto así pero a veces cogiendo el plato y ya se fue todo.

**Ya ustedes han hablado de algunas más cosas, entonces ustedes también antes compartieron algunos problemas en el medio ambiente que se produce en el aire, en las calles, en el agua, en las casa, todo lo que está alrededor de nosotros es el medio ambiente ok, entonces ustedes compartieron algunos problemas que ustedes ven en nuestra comunidad y algunos problemas en el medio ambiente de lo que hemos mencionado y lo voy a poner aquí también pero eso para empezar la conversación algunos mencionaron que hay:**

1. **Mucha basura**
2. **El humo es un problema cuando personas queman basura o el humo de los cigarrillos**
3. **Hay muchos mosquitos y moscas y es un problema en el medio ambiente**
4. **Hay muchas aguas acumuladas ahora que estamos en tiempo de lluvia y que hay mucho lodo.**

**Ustedes mencionaron que esos son algunos problemas aquí en nuestro barrio, quiero escuchar con el grupo aquí si ustedes pueden hablar un poquito más de esto o lo que ustedes ven en el medio ambiente.**

-Unas de las problemáticas de este barrio es que se acumula basura porque el camión no viene frecuente.

**Ok.**

-El camión de basura puede durar hasta meses y no entra aquí a [este barrio] por eso es que se acumula basura en el barrio.

Ok.

-A veces esa basura tiene materia fecal y no la botan a tiempo por vía del camión, pero nosotras las personas debemos de estar bastante consiente respecto a eso, ósea que si sabemos que los camiones no vienen frecuentemente, no echar la materia fecal ni nada eso en la basura porque eso daña el medio ambiente.

-Y los pampers (*pañales desechables*).

**Y los pampers también. La mayoría de personas que hacen con la basura?**

-La queman.

-No--hay gente que la queman y otras que la tiran en la calle o en la caña.

-Si en el camino en la carretera dañando el medio ambiente.

**Entonces usted dijo que [el camión] no viene frecuente.**

-No, el camión no viene frecuente, si viene el sábado puede durar hasta un mes y no viene a recoger la basura.

-Perdón y si ellos vienen por ejemplo este sábado nada más llega de la antena hasta el colmado de ahí. Entonces se llena y se devuelve y puede durar dos semanas más y no viene. El caso es que están dañando la comunidad entre nosotros mismos, no es como la gente que vive en el barrio que busca solución a ese problema. Nosotros por ejemplo no tenemos el ayuntamiento para que vele por la comunidad porque con toda esta lluvia tienen que haber una junta de vecino o alguien dirigente que den vuelta a ver cómo está el agua porque nadie puede salir. Si un niño está enfermo, no puede salir y si hay una mujer embarazada tampoco puede salir porque todo está lleno. Ese es el problema que está adentro de la comunidad, entonces para ponerse a ver las cosas que están pasando por ejemplo el humo del tabaco o el humo de la basura en el aire tienen que empezar a ver adentro de la misma comunidad--como tiren la basura y como las personas hagan la limpieza de la comunidad. Por ejemplo si yo voy donde el vecino, a vecino hay que limpiar el solar dice “mira, vete--que ese es mi solar” y no entiende que tiene que limpiar el solar, porque si limpiamos los solares y ponemos zafacón porque sabemos que la [camión de] basura viene cada ocho día no hubiese basura en las calles.

-Ósea yo entiendo que deberíamos de reunirnos todos no específicamente hacer huelgas, pero para hablar con alguien para que nos acuda respecto a eso porque esto también es un barrio que también necesitamos la higiene.

**Y, ¿Cómo esta el asunto de la basura y de no venir el camión, y el asunto de quema de basura--como afecta la salud de los niños? Como está relacionado eso?**

-A medida de que la persona el camión no viene a botar la basura que pasa que la persona llegan a un estado que se desesperan y dicen bueno tengo este lote que no lo aguanto, sea porque haya más de una cosa dentro, entonces resumen a quemarla, entonces hay personas que no son consiente y piensan vamos a poner esto aquí aparte de que el humo se expande y que pasa...Por ejemplo queman una basura aquí aunque yo vivo de esta casa a aquella el humo va y afecta a mis hijos.

-Claro.

-Y también cuando hay un patio lleno de basura o cualquier cosa hay también se puede acumular mosquitos y se puede acumular parásitos. Después del mosquito el dengue.

**Y, ¿Tenemos mucho dengue aquí o chinkungunya?**

-Aquí no se sabe lo que hay aqui--lo que hay es mucho mosquitos.

-Moscas y mosquitos.

-Hay unos solares que están llenos de hierbas que lo dueño no quieren limpiarlo, al frente de mi casa yo tengo dos solares que están llenos de hierbas y esos mosquitos están que no me dejan dormir.

-Y en los sanitarios como al frente de mi casa, hay un baño y uno no puede coger una silla para sentarse porque no tiene tubo y no vale la pena llamarle la atención ni decirle mira a ver cómo se puede poner el tubo y atrás de mi casa hay hoyo y tuvimos que poner planilla de zinc hasta allá arriba porque si no podían entrar y decirle a las persona que vamos hacer una limpieza para quitarle ese basurero porque la limpieza es muy importante.

-También otra cosa el barrio cuando llueve uno no se puede mover para ningún lado porque está demasiado feo por el lodo.

**¿Por el lodo?**

-Sí, se pone feo.

**Bueno, ¿Hay más problema que no hayamos hablado? Hablamos mucho ya de la basura, del humo y de algunos mosquitos y moscas. Hay otros problemas en el medio ambiente que no hemos mencionado?**

-Bueno respecto al agua acumulada.

**Ok.**

-Eso es otro problema que también afecta, cuando dejamos por ejemplo una llave abierta y se vuelve un poso ya eso es agua acumulada puede crear mosquitos, el parasito y luego el mosquito, entonces eso nos puede afectar a todos, el mosquito a veces te deja a ti y se va hacer daño a otra parte y no puede dañar a nosotros mismo.

-También el agua estancada--estancar mucha agua y dura mucho día si tú la vas a estancar debes de usarla aunque sea en el trascurso del dio ósea hoy y mañana o pasado porque si la deja bastantes día, ya tu sabes, mosquitos.

**Algo más? Entonces gracias por compartir todas esas opiniones fue muy interesante escucharlas y han dicho muchas cosas interesantes, bien pensando ahora en algunos problemas de salud y como están relacionadas al medio ambiente y problemas que vemos aquí en nuestro barrio, vamos a pensar en el futuro y cómo podemos seguir adelante para abordar estos problemas como individuos, como vecinos, como parte de esta comunidad. Si fuera muy fácil solucionar todo, no estuviéramos aquí, pero yo quiero escuchar de ustedes cuales son algunas intervenciones, cambios o cosas que la comunidad puede hacer para mejorar algunas cosas en el medio ambiente para mejorar la salud de los niños.**

-Yo entiendo que como personas adultas que somos, debemos usar más el dialogo con todas las personas y mucho más con las personas que tenemos cerca. Cuando tenemos un problema referente al medio ambiente, debemos hablar con todas las personas y la autoridad, con toda la persona que nos puede ayudar como el vecino…porque decimos que no podemos hablar con la vecina y no está haciendo daño que vamos a esperar que estemos internos o nuestro hijos, cuando nosotros mismos estemos internos entonces vamos a hablar con la vecina. Debemos ser inteligente y hablar con los demás, aprender a hablar con los demás porque no es justo que nosotros comamos normal y no podamos hablar normal. Todo no puede ser pleito, uno no puede pensar que para yo hablar con ella tiene que ser en una guerra para no entendernos o que no somos? Seres humanos? Yo entiendo que somos seres humanos como no podemos entender que todo tiene que ser una enemiguesa específicamente. Si yo entiendo que le estoy haciendo daño a ella otra persona me puede decir, “Mira, tú le estás haciendo daño a [esa persona], yo tengo que buscar el medio porque hay una basura acumulada hay un mal olor a materia fecal que le está haciendo daño a ella,” y si yo entiendo que soy un ser humano, yo debo de entender porque tenemos raciocinio y es para entender porque todo no puede ser enemiguesa hay que buscar el medio también y saber dialogar con la demás personas para no hacernos danos a nosotros mismos porque por temor de no hablar no hacemos daño todos.

-Yo quiero darle una corona a la palabra de [ella], porque para hacer lo que dijo [ella] como seres humanos porque parece que no es todo el mundo humano, ahora si primeramente con los ojos abierto buscan un par de persona sean diez o quince personas que sepan que somos humanos y enfrenten esa persona y le digamos que necesitamos la limpieza de esta comunidad. Hay que hacerlo antes de enfrentar a los que no son humanos (unirnos) y ponernos hacer ese trabajo. Entonces cuando empecemos a trabajar en la comunidad, si no podemos enfrentar a las personas que no son humanas pueden buscar la fuerza de la autoridad para hacerlo específicamente--porque yo me acuerdo de un señor que tenía un solar lo que limpiamos, y lo llevamos al tribunal y dice el fiscal pero tu tiene que pagarle a ese hombre porque te hizo un buen trabajo.

-Jejeje claro.

-Ya hay se ve que esa persona no era humano porque yo le hice un buen trabajo y honradamente ya me hacen eso otra vez y le digo vamos para el tribunal para que el fiscal diga tienes que pagarle a ese hombre o te voy a meter preso lo que hay que hacer aquí en el barrio empezar a unir como dice ella a ver si hacemos algo.

**¿Y qué piensan ustedes?**

-Eso es así. La unión hace la fuerza.

**¿Cuáles son algunas barreras de esas o cosas que hace la unión es difícil porque a veces no es muy fácil hablar con los vecinos?**

-A veces nosotros mismo somos lo que lo ponemos difícil.

**¿Cuáles son las otras ideas que tiene ustedes como para unir la comunidad?**

-El temor de hablar hay que dejarlo.

**Si--eso es una barrera?**

-El que equis persona diga sin tratarla...fulana es muy difícil, pero nunca la trataron por el simple hecho de que fulana es muy difícil, que pasa? Yo me llevo de eso y nunca me dirijo a fulana me lleve de la especulación de aquel que nunca ha tratado aquella y nos detenemos allí. El daño se propaga, se propaga y se propaga y nunca tratamos de romper esa barrera de involucrarnos, no, pero ven acá, yo tengo que saber qué es lo que pasa, “oiga vecina está pasando esto y esto vamos a cooperar.” Hay personas que tienen ese don, hay otras que le encantan hacer la cosa detrás del otro, y yo no estoy de acuerdo con eso. Si somos vecinos y va a pasar algo va a ver una limpieza si nos juntamos las dos se hace mejor el trabajo. Creo yo así.

-A veces hay personas que dicen “fulano esta quillado.” Por un ejemplo: si yo fumo y pasa uno fulana “vota ese cigarrillo” esa no es la forma de decirlo, tiene que decírmelo que yo pueda entender de que me va hacer daño, no nada más me hace daño a mí que lo estoy consumiendo. Pero también la falta de comunicación y la falta de relación humana es que hace que las cosas se compliquen y esa es una de las cosas también.

**¿Cuáles son algunos esfuerzos comunitarios, o sea, que podemos hacer, que tal vez sean posibles en el barrio para mejorar uno de esos procesos, una cosa que podemos hacer?**

-La fuerza de una junta de vecinos si hay. Así se hace más fácil, pero si no hay, o sea si hay lo que no están, no califican, o sea, no tienen tiempo para eso, la fuerza de la comunidad en millón de veces si hay…y si no hay, la comunidad está en el suelo.

**Y entonces, ¿Cuáles son las opiniones de ustedes, las personas que viven aquí, creen que vale la pena, hablar con la junta de vecinos para mejorar algo? O qué?**

-Mira, yo puedo entender que las personas han confundido también una cosa, en cada barrio, como quien dice allí una cabecilla, quien pueda dirigir un poquito la comunidad, pero la junta de vecinos somos todos. Somos todos. No debemos de esperar que una persona diga: “bueno aquí se va hacer lo que yo diga,” no, no, no, aquí se va hacer lo que todos digamos, porque si nos reunimos entre todos y acordamos en que aquí no se va a quemar la basura, no vamos hacer tal cosa, entre todos. O que yo venga y diga: aquí nadie va a quemar la basura, nadie lo va hacer, nadie lo va asumir, aunque a mí me destaquen como presidente de la junta de vecinos, todos nos podemos de acuerdo para evitar lo que nos hace daño. Como en una reunión, por ejemplo, se puede hablar de que la quema de basura hace daño en esto y esto y nos vamos a entender entre otras cosas. Pero si esperamos junta de vecinos nunca vamos a echar para adelante, nunca. Porque la junta de vecinos no le duele el barrio, es nosotros es que nos duele el barrio, no la junta de vecinos--porque una sola persona, que es lo que va a decir: bueno yo lo que puedo hacer es arreglar mi frente, si me traen un camión de materiales, que me importa a mí, no me importa las demás personas. Nosotros tenemos que dejar de pensar en nosotros mismos.

-Y otra cosa es: como dice ella, que en cada junta de vecinos hay que haber una cabecilla, ahora si hacemos esa actividad o las puertas abiertas, o algún voluntario o voluntaria, ayudar a formar un equipo de vecinos “ ayudar al vecino,” y podemos trabajar voluntariamente por la comunidad. Entonces si usted viene de allá del Barrio George o Los Jardines, encuentra el barrio limpiecito, no hay polvaso y entonces esa gente que viene a compartir con todos nosotros y todo ese lodo, entonces les damos valor. ¿Usted me entiende verdad? Si por ejemplo necesitamos chapear y, ¿cuánto cuesta un machete?, como doscientos pesos por ahí, voy para donde [ella], [ella] no tiene, pero ella puede darnos vasos de agua, ahora si hacemos ayuda de alguien que podamos comprar: limas, los machetes y se decida a dárselos a la gente del barrio.

-Yo me acuerdo que [otra persona] y yo hicimos una actividad de chapear y [otra persona] coopera con nosotros y [ella] hablo con [otro] y el cooperó con nosotros con la lima y el barrio está limpio. Lo que pasa es que esto no tiene seguimiento.

**Algunos de ustedes mencionaron que si personas se reúnen en dar conocimiento sobre la quema de basura, que a lo mejor no es lo mejor para la salud de los niños, es muy difícil parar de quemar la basura y más cuando es la única alternativa, ¿Cuáles son algunas alternativas, por ejemplo, para dejar de quemar la basura?**

-¿De quemar o no quemar?

**Sí, una persona dice: no debemos de quemar la basura, porque tenemos niños con asma y eso es un problema, seguro que muchas personas van a estar de acuerdo con eso con la salud de los niños pero, ¿Hay alternativas que quemar la basura?**

-Yo diría que sí, buscar sacos y saber que echar en esos sacos.

**¿Y si no viene el camión?**

-Quemarla. *(muchos están de acuerdo.)*

-Yo diría no venir nunca, yo diría no venir nunca, porque donde está el problema es que saber echar en la basura, porque hay personas que echan: ratas, materia fecal, como dije ahorita, siendo cosas que surgen mal olor.

-Llevarla a un sitio donde se pueda quemar la basura.

-No quemen basura, eso es lo mejor. Nos podemos de acuerdo con el ayuntamiento y ya sabemos que el camión viene cada ocho días, viene todos los sábados o todos los domingos y si el camión viene lleno, si el camión viene a las una de la tarde (01:00 PM), o a las siete (07:00 AM) de la mañana, se limpia el barrio inmediatamente.

-Porque en antes entraba el camión y ahora no entra aquí temprano.

-Entonces el primer cambio si podemos hacer para una oportunidad de no quemar basura es que el ayuntamiento tiene que estar puntual [el camión].

-Claro, votarla en la calle y como quiera contamina el medio ambiente, porque todo ese humo viene para acá.

-Ahí en el camino, esos potes cuando se llenan de agua y esas latas de picantinas *(sardinas enlatadas)* también se llenan de agua y se crían los mosquitos.

-Otra alternativa también que yo considero es: reunirnos todas las madres, e irnos directamente al ayuntamiento y hablar con el jefe y hacerle saber que nosotros también tenemos derecho—[nuestro barrio] también tiene derecho a estar higienizado, claro. El mismo puede venir a observar y no es que venga hoy y mañana no, venir todas las semanas que es lo lógico. Él estaba muy afanado cuando él quería que le dieran votos, él vivía aquí metió, dizque que iba hacer esto y aquello y tan pronto gano, jamás volvió.

-Y no es eso, sino que ellos dicen: no vamos a entrar porque hay bache (lodo), aja, ¿a quién le corresponde arreglar las calles? Es al síndico, pues arregla las calles para que pueda entrar el camión.

-Así mismo, mi opinión es que cuando lleguen las elecciones es que quiten al [sindico] del poder, porque lamentablemente él no ha hecho nada para Consuelo.

-Nada.

-No, porque lo que él dice es que en esa comunidad hay demasiados Haitianos.

-jejeje, pero el primer Haitiano es el, él no se ha dado cuenta de eso. Asi mismo.

-Hay barrios en el que el camión de la basura pasa todos los días porque ellos pagan una cuota, ¿no verdad? No sé si en este barrio aceptara pagar una cuota, aunque sea dos veces.

-Yo pagaba mi cuota de basura, inclusive yo hasta tengo mis facturas, tengo hasta recibo de la basura que yo pagaba yo pagaba veinte pesos ($RD20.00).

-Yo también.

-Una vez iba a entrar un camión lleno de yuca para acá adentro y el dizque no se iba a meter porque decían que no había votos y el camión que iba a dar la yuca se devolvió. jejeje.

-Por ignorantes no entraron, porque los haitianos somos personas también, esas son personas ignorantes, porque aquí muchos haitianos votaron por él, votaron por [el síndico].

-Sí, porque hay muchos que tienen cedula.

**Yo sé que a lo mejor muchas cosas tienen que ver con lo político, tiene relación con lo político. Hablando de los esfuerzos comunitarios de los que están dentro de nuestro barrio, podemos votar para dar poder a alguien que pueda ayudar, y ¿Cuáles son algunas cosas que, ustedes ya dijeron muchas cosas buenas en las que podemos trabajar en la comunidad, cuales son algunas cosas que ustedes piensen más, puede ser cualquier idea, para mejorar más cosas de la salud o cambio en el medio ambiente?**

-Tengo una idea en la mente, no sé si es primeramente, un tema de la salud--necesitamos en la comunidad: primero auxilios, porque si en [este barrio] hay enfermedades. Yo estoy oyendo mucha gente diciendo los que tienen niños de [este barrio] cogen lucha en Maternidad, si porque ellos se van y reciben sus medicinas, o una móvil. Mi niña tiene diarrea y está vomitando, lo último de lo que él está hablando es del programa que tiene Ramona y así cogen menos lucha en la Maternidad, porque a veces son las diez o las once de la noche y uno tiene que ir para Maternidad, no vete para el Musa y si es un vago no tiene para pagar un moto concho para ir al Musa y si llama a un vecino y vamos a coger para Maternidad y cuando viene a ver uno con un niño en los brazos para el Musa y si aquí hubiera la oportunidad de los primeros auxilios eso sería un éxito para la comunidad, hasta que abran el centro de salud de allá para la comunidad y otra cosas sobre el tema de la salud, primeramente sentarse con el vecino, plantearle algo que le sirva para la salud de los niños, es que es bueno. Preguntarle al vecino ahora que salga a buscar par de gomas, barrio abajo y así ver cómo están todos los callejones, ver donde se pueden echar un viaje de material, niños que van para la escuela o alguien que va a limpiar zapatos y entonces con todo eso, vamos a suponer que hay niños durmiendo y van para la escuela y pisan esa agua, pueden adquirir una enfermedad y ya como dijimos ahorita: un tanque de agua que dura varios con agua, puede dar el dengue, al igual un pozo de agua puede traer todas esas cosas y primeramente en el barrio puede haber más enfermedad atraves de la basura y por la falta de unión.

**Gracias.**

-Otro tema que voy a tocar es el de la luz: aquí hay muchos alambres que son de aluminio y cuando llueve los niños no pueden salir afuera por miedo a que se parta un alambre de eso ya que es peligroso ese alambre de aluminio, se puede caer y ya no hay para nadie.

**Hablando del tema de la basura estoy curiosa, ¿hay algunas personas que ya reciclan cosas, o sea toman botellas de vidrio o cosas de plásticos y venderlos para reusar, comprar o no?**

-¿Aquí en el barrio?

-Ahí hay un señor que se lo lleva.

-Pero no todas.

-El las elige las de cervecita y de cerveza presidente, y las de Vino La Fuerza él no la compra.

-Y las de Malta Morena también.

-Recicladas él no las compra.

-Ella se refiere a las cosas recicladas que sacan de la basura, a eso ella se refiere. Las fundas de café nada más, las fundas (X) nada más, las botellas plásticas nada más, y así sucesivamente.

-Los muchachitos del barrio desde que ven una silla rota ello lo venden y los potecitos también, para cuando pasa los camiones viejos ellos los venden también.

-¿Compran los potes de refrescos?

-Sí, los mismos niños del barrio se encargan de limpiar el barrio, porque todas esas sillas, cuando votan una silla rota la cogen y la venden, los potes de refrescos, de Kola Real, todos esos potes también lo venden. Una vez un señor, no sé si era loco que estaba pero recogía los potes.

-Pero mi abuela recogía los potes y tuve una vez que votarlo, porque ya no había donde ponerlo y ¿Por qué no lo vendía? Una bolsa de potes viejos y después tuve que votarlo en el camión de la basura, como más de cinco sacos de potes viejos.

-Tu sabes donde vivía ese hombre, quién es ese hombre, a él una vez lo encontraron al lado de la casa del soñador una vez.

-Si, él lo recogía para el soñador y la mujer de él, no sé si lo vendió, ¡pero un saco como de este tamaño!

-Sí, pero las sillas plásticas yo sí sé que ellos la venden.

-Porque yo vi, cuando venía de la escuela, como del lado de la casa del señor, yo vi que venían unos camiones, con muchas botellas plásticas que llevaba y mi abuela junto más de ocho sacos y nunca vi que lo vendió, lo fuimos quemando y votándolo de chin en chin, eso es lo que hay: cuando no viene el camión, hay que quemar la basura.

**¿Es difícil hablar con los vecinos sobre sus preocupaciones del medio ambiente o fácil o cual es la experiencia?**

-Hay vecinos que entienden, pero hay otros que cuando uno le dice una cosa ellos piensan que uno lo que esta es engañando y lo mejor a veces es no meterse en eso.

- Y hay otros en los que uno le habla y meten otro tema, sí.

-Y le dan a uno su buen boche.

-Y es mejor uno no meterse en eso.

**¿Entonces eso es una barrera de reunir?**

-Eso es el mal vivir, vecina me preocupa.

-Todos somos vecinos, [ella] es mi vecina y ella vive más lejos y es mi vecina.

-Él tambien es tu vecino.

**Bueno hay algo más que no hemos discutido que quieren compartir en este grupo?**

-Eso es otra cosa la energía eléctrica, que se va horas, anteriormente se iba a las dos y venía a las siete, ahora viene a las ocho se va a esta hora, y llega a la hora que todo el mundo está durmiendo cuando uno la necesita. Eso no es justo.

-Pero no, tú sabes que la noche es larga y como esta en día ese es el problema y es mejor que no haya.

-No creo que es mejor que hay.

-Nada más tirando tiro.

-Yo estaba afuera y cuando vi eso.

-O pero muchacha dos tiros.

-Y cuando fue eso.

-Anoche y bien sonado, pero dos tiro, mira, yo tenía la puerta de adelante cerrada y la de atrás abierta y agarre la niña y la cerré.

-Y que tú hacías a esa hora afuera.

-No eran las nueve no estaba tarde, mi amor.

-Bueno ‘manita yo a esa hora estaba acostada.

-Tú crees que yo con una niña chiquita voy a salir.

-No yo salgo y estoy ahí afuera en mi patio.

-Y es verdad ella puede estar en su patio su patio es su casa.

**¿Los sanitarios son un problema aquí en este barrio?**

-Bastante. Si.

-Problema porque por ahí por mi casa hay un mal olor a materia fecal que no se aguanta. Muchas personas viven alquiladas en casa y no le hacen baño, tienen que poner los niños a evacuar y que hagan hoyos de verdad yo pensaba que nada más eran los gatos que hacían eso.

-Y los niños lo hacen también. Las personas adultas deben ser responsables porque yo no le alquilo una casa a nadie que me la proponga, lo primero que yo tengo que ver es el baño, no importa que yo viva estrecha pero un baño tiene que ser lo primordial. Las madres aceptan vivir en una casa sin baño y ellos mismo le hacen un hoyo para poner los ni nos a evacuar y después taparlo. Eso es algo terrible.

-Parece como que no le afecta a las madres como que no tienen nariz cuando ensucian los patios y cuando llueve, ese mal olor sale que uno no lo aguanta.

-Hay yo no tengo ese problema. Yo soy la única que usa el baño. Es un problema de salud pero es importante porque si no hay salud no hay nada, ojala que Dios nos permita calmar los problemas de salud. Esta comunidad tiene problema intocable que todo el mundo tiene miedo de tocarlo, pero vamos a ver si Dios nos permite que arranquemos con la salud y con la quema de basura, pero el tema de la salud es importante.

-Pero la salud… el bajo a materia fecal, la basura quemarla, el agua estancada todo eso afecta la salud, que si uno no evita todo eso la salud no puede estar normal.

-Cuando hablamos del tema de la luz, pero no tenemos agua, no piensan que en esta semana viene y la otra no, hay tiempo en que no hay agua, no es que es así en toda parte, por cristo viene hay un tubo de agua pero es como de trecientas personas pero por aquí son solo tubitos de agua y cuando viene a ver yo tengo que salir a buscar agua porque aquí no hay y fulano tiene agua ella que vive a dos esquinas tiene que cargar agua por la rejilla para ella tener agua y nosotros tubitos.

-También en la materia fecal tu sabes que hay muchos cólera ahora es que está un poquito controlado, pero a través de eso es que inicia el cólera ya las personas no entienden que la materia fecal produce cólera y ellos no entienden eso.

**¿Creen que es falta de conocimiento que tienen algunas personas en el barrio que no conozcan las conexiones entre salud y el medio ambiente?**

-Hay muchas personas que no tienen conciencia, porque si muchas personas tuvieran conciencia no estuviera pasando todo esto, como dije horita muchas personas no tienen su hogares aquí, muchas personas viven alquiladas y no es justo una gente alquilar una casa sin no tener donde hacer sus necesidades que parte de la vida nos dio. Eso es falta de conciencia.

-Y muchas personas saben muy bien que están y tienen conocimiento que eso trae enfermedad y que hay que tratar ese problema de limpieza.

-La pregunta que usted hace sobre el tema de la salud es que no hay un permiso para que uno pueda entrar en el baño de la persona para controlar como está el patio, porque atrás de mi casa usted puede ver hay un séptico y eso lo que parece es una cubeta sin tapa, porque toda esa moscas.

-Eso es verdad.

-Y hay otra casa usted va por el callejón de esa casa y ve trapos, zapatos, chancletas y cubetas todo hay tirado--palo, alambre, todo hay ahí. Debajo de todo eso, puede ver ciempiés, puede a ver hasta ratoncitos. Entonces en el tema de salud, yo tengo mi patio bien cercado y tengo mi portón cerrado y se puede hacer cualquier cosa ahí, por ejemplo puedo buscar una funda y los vecino míos mirar y tirarla para ahí abajo.

-Eso si es verdad.

-Eso se hace, para tocar el tema de salud y de limpieza hay que ver y hablar con esa persona. [Ella] me permite ir a su casa y cuando llegue a su casa dígame que usted piensa de la limpieza y de todo eso hay, no pero esa es mi casa y decirle límpialo que te sirve a ti y al otro, no, no, no déjemelo allí, entonces usted ve a esa persona y no hizo la limpieza. Se llama la autoridad para que haga su acción porque la persona están haciendo un daño, porque por ejemplo a mí no me está haciendo un daño, pero se lo está haciendo a otro porque un ciempiés sale del callejón de mi casa y se va para donde el vecino y después va para donde otro. Cuando viene a ver si un ciempiés pica a un niño, y el padre, vamos a suponer sale y deja al niño cerca de la casa solo, y llega un ciempiés y el viene por la tarde o por la noche y dice tiene hambre o tiene calor y le da galleta y trompa come, come y fue un ciempiés que lo pico entonces cuando lo lleva para el hospital ya era tarde, y porque, por un egoísta que dejo toda esa basura hay con trapos como que no se votan. Los trapos se votan, no lo guarda allí para hacerle daño a la comunidad. Si yo fuera una persona con autoridad, yo no metiera a todas esas personas presas, yo le hablara con calma y con amor--vamos hacer esta limpieza. Ósea otra gente jodona y le digo: mira vamos hacer una limpieza hay y lo van hacer de una vez, de una vez hacen la limpieza si voy yo hacer la limpieza de la comunidad, pero otro dice: vete que es lo que es lo tuyo.

-Si es verdad, pero puede venir un borrachón de ahí de Hato Mayor.

-Se sientan a escucharlo.

-Pero para arreglar la comunidad es muy difícil.

-Porque no se ganan la confianza de la personas.

-Pero hay en el cinco ellos creen en mí, porque si yo voy para donde [ella], vamos a tumbar es mata, [ella] dice buen, yo soy una hembra no puedo cortar esa mata, pero busca alguien y vamos a darle algo, pero hay gente que dicen no, déjame mi mata ahí.

-Eso es así.

-Pero es lo que dice [ella], tu tiene que saber con quién tú hablas.

-¿Cómo?

-No como, si usted vive cerca y puede ir con cinco fundas en la mano, y dársela a una persona, mire amiga, tome, tu eres la mala por la funda y como tú vas es con quien, es con quien porque tú puedes ir con un santo y no con una persona porque se te suben encima.

**¿Qué piensan los demás de esto?**

-Yo entiendo que es como tu aborde a la persona en que momento y en qué tiempo tú vas a abordar a la persona, porque algunas personas de aquí no es todos los día que amanecen con el pies derecho. Entonces uno debe y tratar de conversar a las personas de manera amiga--mire ese árbol, si llueve o hace un temblor, ese árbol le va a partir la casa en dos, y te vas a quedar en la calle ósea que hay que hablar.

-Con calma.

-Que hay que hablar de una manera con la otra persona para que puede recapacitar. Las personas de aquí viven como que no entienden de las demás cosas y se hacen daño ello mismos y a los demás.

-Lo que iba a decir que es viendo algunas casos de la que se hablaban, que están pasando es por falta de comunicación, ósea falta de orientación, porque hay personas que sí que si tiene un árbol en su casa ven que quizás le sirve para la sombra, pero si viene una persona y lo orienta y le dice en que le puede afectar quizás van a decir que sí.

Entonces a veces es falta de orientación.

**¿Algo más?**

-Este hay otra cosa porque la personas de aquí ósea no sé si es que tenemos algo que creemos que todavía somos niños, que la orientación hay que dársela como medicina para que puedan entender que esta bien para ellos mismo. Es algo que todo los días hay que hablarle a las personas como que es una escuela para los adultos, que a pesar de que sean bachilleres o como hay muchos en octavo grado hay que estar hablándole todo el tiempo la mismas cosa para que no se le olvide. Es obligatorio dar orientación con respecto al medio ambiente todos los días.

**Alguien tiene más para compartir? Bueno, entonces muchísimas gracias a ustedes por su participación.**
