# Supplementary material for: Talking trash: Perspectives on community environmental health in the Dominican Republic
Source: PLoS One. 2021 Mar 29;16(3):e0248843. doi: 10.1371/journal.pone.0248843 (PMC8007031; doi:10.1371/journal.pone.0248843)
Supplement: S6 File — (DOCX) [file pone.0248843.s006.docx]

***(Introducción)…***

**…Ustedes compartieron sus opiniones sobre problemas de salud más comunes… (*Explicación*)**

1. **La gripe**
2. **La fiebre**
3. **Diarrea/Vómitos/Dolor de Barriga/Amebas**

**Vamos a discutir en grupo.**

**¿Qué opinan ustedes de este listado, hay otras cosas que debemos mencionar que son bien frecuentes aquí en [este barrio], o que causan muchos problemas con los niños?**

-Bueno, después de la ameba hay más como el tricocéfalo que son otros paracitos que no son ameba, y como usted dice la fiebre, a veces a un niño le da fiebre cuando estan jugando le da fiebre y muchas cosas más.

-Andan muchas enfermedades, el niño mío la otra vez le subió una fiebre así de repente y también de repente esos vómitos y la diarrea.

**¿Hay muchos vómitos y diarrea aquí?**

-Sí.

-Yo trabajaba donde una señora y había una niña y se comió su desayuno y se sentía mal y después esa niña disparó todo, todo vomitando.

**Entonces porque piensan que hay tanta ameba o tantas diarrea y vómitos con los niños?**

-Bueno, no sabemos lo que está causando eso, pero a veces yo creo que es porque uno le da un agua y cuando uno se están bebiendo eso pasa. Hay chiquitos, pero hay grandes también que pasa eso.

**Hay otras cosas que talvez afectan a los niños y a lo mejor son más severas, ósea que no afectan con tanta frecuencia a tantas personas, pero cuando una niña tiene eso es muy grave o puede complicar.**

-Bueno donde hay agua sucia y mal olor, eso puede complicar.

-Y a veces si hacen mucho contacto con la tierra también, porque a veces los niños vienen y al momento uno no la baña, lo digo por la mía, al momento del baño están recogiendo piedras y echándolas en vasito o en la boca, y cuando la baño le echo un chin de manitas limpias y le lavo las manos, o le echo un chin de shampu también porque cuando yo vengo a dar la vuelta, ellos están otras vez con las mismas piedras.

-Y ese aire contaminado, mire--esos cambios de temperatura mire, de ahi viene todo esa gripe y ese apretamiento en el pecho.

-Se aprietan mucho.

**¿Se aprietan mucho?**

-Sí.

**Cuéntame más de eso, ¿hay muchos niños que se aprietan o que tienen dificultad para respirar aquí, a veces?**

-La mía cuando tiene problemas de la gripe, ella siempre presenta eso dificultad para respirar, a veces siente un miedo de madrugada y me dice: mami me duele el pecho.

**¿Y qué piensan ustedes, cual es la razón más común para llevar a un niño al médico?**

-Bueno, las cosas que más o menos impulsan a uno a llevar al niño al médico: cuando el niño tiene vómitos y diarrea, hay que llevarlo al médico o sino se deshidratan y pueden perder bastante liquido o hasta morir, porque imagínese el cuerpo sin líquido no se puede pasar de líquido pero tampoco menguar.

**¿Y qué piensan los demás? Entonces pensando en eso: en vómitos y diarrea, dolor, y fiebre, vamos hablar de algunas cosas que algunos mencionaron durante la discusión anterior, entonces otra pregunta fue “¿Cuáles son algunos problemas en el medio ambiente, osea el aire, el agua, la tierra, las casas, los edificios, todo lo que está alrededor de nosotros, en nuestra comunidad?” Entonces vamos hablar de algunas cosas que ya ustedes mencionaron, que a veces son problemas que ven aquí en este barrio. Lo que ustedes me dijeron:**

**1. Mosquitos y Insectos**

**2. Basura**

**3. Quema de Basura**

**4. Letrinas**

**5. Muchos motos**

**6. Calle deteriorada**

**O sea, ¿qué piensan de este listado, cuales son las cosas en el medio ambiente más preocupantes, más problemáticas?**

-Bueno, podríamos decir: mayormente los insectos y los mosquitos, porque en cuanto a basura quizás, aquí no tenemos un lugar que haya tanta basura, porque siempre pasa el camión o si no hay vertederos que las personas la tiran y así no la tienen dentro de la casa. La quema de la basura también, digo yo es un poco por aquí, porque el camión pasa.

-Porque si el camión pasa, no sé porque están quemando la basura, entonces no solo es basura normal sino que también son papeles y eso no le conviene al medio ambiente, porque lo contamina, los mosquitos, muchos mosquitos. Y la basura, como ella dice, el camión pasa cada seis y siete días, hay veces que da dos viajes a la semana. Pero también las letrinas, algunas personas que no tienen baño, pero tienen la letrina ahí y cuando eso desemboca mira uno tiene que dejar de comer o algo, tiene que dejarlo eso es algo desagradable.

-Las calles, cuando nosotros tomamos un motoconcho y nos vamos, usted siente todos los dolores aquí detrás, con tantas piedras y tantos hoyos que hay. Otra cosa, cuando uno se pone unos zapatos nuevos, a mí lo que me duran son dos días, mire, de una vez se me pelaron y yo no sé qué hacer. Se me dañan las zapatillas y había posibilidad de que la iban arreglar, pero nada más fue una bulla (aguaje) que hicieron allá.

**Gracias por compartir eso. ¿Cuáles son las experiencias de los demás acerca de la basura, por ejemplo?**

-El camión pasa.

**¿Muchas personas queman la basura?**

-Bueno, a veces cuando el camión no pasa, como un ejemplo: que dura como ahora que en estos días vino dos veces repetido, porque duro casi quince días sin pasar.

-Entonces se aprovechan y tiran los papeles del baño y ese mal olor, ya usted sabe. Ese a veces como que afecta, le hace mucho daño a uno.

**Y hablando de mosquitos e insectos, mencionaron que hay muchos aquí. Cuéntame más de esto.**

-Esos mosquitos e insectos, principalmente los mosquitos, eso podría ser causa también de los mismos árboles que hay mucho, en el barrio hay muchos árboles.

-Y de noche es que pican esos mosquitos. Eso es por las matas (arboles).

-Vamos hablar de esto: del motor. Ustedes saben que hay muchos motores en la calle y también en el barrio y hay muchas personas que son motoconcho y algunos son tranquilos pero hay algunos que por hacer su firifolla (piruetas) otros que cuando aceleran ese motor y ese humo que sale es otra cosa. También eso es toxico y es desagradable para la salud.

-Y (el humo) se le va a los pulmones.

**¿Y los motores es un problema de accidentes aquí o no?**

-Sí, muchos accidentes.

**¿Aquí en este barrio o no?**

-No.

-En el barrio no.

-En Consuelo.

**¿Hay mucho dengue aquí o chinkungunya o enfermedades de mosquitos?**

-Había mucha aquí.

**¿Ustedes conocían muchas personas que la tenían?**

-Uff, el barrio entero jejeje.

**¿El barrio entero?**

-El barrio entero.

**Y ustedes, solo por curiosidad durante, ustedes saben que hace dos meses atrás, que había mucho chinkungunya en el país y todo. ¿Fueron algunos cambios aquí en el barrio o mejorar eso, o algunas cosas o personas vinieron a educar? ¿Qué ustedes hicieron como comunidad para mejorar esa situación?**

-Un solo día paso un camión fumigando en las calles, pero los métodos que nosotros siempre tenemos es: no dejar agua acumulada, estancada, y las que vamos a usar tenerla tapada y con un chin de cloro.

**¿Pero siguen con muchos mosquitos?**

-Ay, sí.

**¿Hay mucho dengue?**

-No. Ahora no.

-Muchos mosquitos, por aquí, por aquí mismo no tanto.

**¿Entonces otras cosas que no hemos mencionado u otras cosas que no hemos comentado, u otros problemas que ustedes piensan que afecta la salud? Ok, hay muchas cosas que mencionado y que son diferentes como problemas de salud, ahora vamos hablar pensando en estas prioridades de la salud o problemas que ustedes mencionaron, pensando en estos problemas del medio ambiente. Vamos a discutir: ¿Qué puede hacer la comunidad, o sea los vecinos, las madres que viven aquí con apoyo de otros lugares?¿Cómo pueden trabajar juntos la comunidad para mejorar el medio ambiente o algunos de estos problemas, con la finalidad de mejorar la salud de los niños, que piensan ustedes? Ha hecho algunas cosas la comunidad para mejorar el medio ambiente o la salud, algunas intervenciones?**

-Yo creo que no.

-Aquí en [este barrio], casi no entra nada. Usted ve que hacen muchos operativos médicos y regalan muchas cosas, pero aquí en [este barrio] no, como que las personas quieren ayuda pero las ayudas no llegan.

-[Este barrio] tiene los ríos.

-Exactamente.

**¿Qué piensan los demás, tienen otras experiencias, otras opiniones? Ustedes son las expertas, viven aquí, pueden tener diferentes opiniones y no pasa nada, todo eso es para escuchar. Que se puede hacer, ustedes en su comunidad?**

-Yo digo que si uno tiene una ayuda, que puede mejorar. Pero si uno no puede, no se puede hacer--digo yo, a ver si se puede mejorar.

**-¿Cómo un apoyo de alguien?**

-Sí, exactamente.

**¿Apoyo de donde o de quién?**

-De una comunidad o persona que pueda ayudar a uno, puede mejorar, pero si no hay ayuda no se puede mejorar.

**¿Cómo pueden lograr esa ayuda?**

-Ir donde una persona, que tenga la manera de ayudar a uno que no tenga y así uno se puede ayudar uno al otro y así sucesivamente.

-Claro, porque hay que comprar el cloro, que hay que comprar una bomba de fumiga, hay que comprar el producto y uno no tiene ese dinero. Entonces yo no estoy trabajando y hace mucho que no estoy trabajando y que deje de trabajar, y de lo poco que consigo no dejo de comprar mi cloro, a veces para fregar y friego toda la orilla de la casa, porque la casa mía está en muy mala condición, la están construyendo, pero adentro esta ya usted sabe. No es fácil. Por las noches, hacen como dos semanas yo eche un tres pasitos (veneno para ratas) y mate unos ratones, así de ese tamaño, jejeje.

-Los ratones son un problema del medio ambiente también.

-Sí.

**¿Eso es un problema aquí?**

-Sí y ahora, yo veo eso sube de la barranca donde están todas esas matas y eso que tumbamos todas esas matas que estaban alrededor de la casa y en estos días subió uno, como que revivió y para donde estoy durmiendo ahora, ahí están subiendo los ratones, ahora voy a ir a ver si hay más tres pasitos más para echarle para ver si se eliminan, así son las cucarachas. A veces si no puedo hacer una cena temprano, me quedo mejor sin comer para no tener que cruzar de aquel lado donde está la estufa, que es donde tengo mi casa donde vivía primero, porque a veces estoy yo muy campante y cuando estoy haciendo mucha memoria, de que no quiero que se me peguen moscas en los alimentos o algo, si pasan de las seis de la tarde me quedo sin comer.

**-¿Muchas cucarachas?**

-Sí, yo he comprado Baygon y trajeron arizolin también, el olor de eso las ahuyenta y no veían nada y al momento de que se me acabo el arizolin y ahora vuelven otra vez, pocas ya no como antes y como una a veces algo me dice mira para ahí, como que siempre mi mente está pensando en esos pájaros (insectos) salgo corriendo jejeje.

-Saben que eso lo que ella dice es una forma de nosotros mejorar nuestro ambiente. Pero de nada nos sirve hacerlo nosotros solo en nuestros hogares, sino unirnos y hablar con nuestros vecinos, para que vayan corriendo la voz. Porque hay veces que yo limpio mi patio y no tiro basura, y no tengo agua acumulada, pero en el patio del vecino si hay y todos los ratones y cucarachas se van de un patio a otro, entonces esa sería una buena forma de nosotros mejora nuestra comunidad.

-Claro.

-Mira, que el vecino pueda, mira un ejemplo ahí, esta todo esto limpio verdad, esas matas de coco que no están en nada que se esconden los mosquitos y los insectos ahí e ir podando todo eso y se ve algo. Ahora más en estos días es que hay que atacar al vecino, porque el vecino no limpia, el vecino lo que gusta eso y nosotros entramos de aquel lado, y toda la basura la tenemos amontonada del lado de atrás, cuando se desplome y entonces cuando las matas se estén secando, agarramos toda esa basura para sacarla para afuera, porque la gente de la basura no la sacan. Hay que dejar que los palos se sequen un poco para echarlo en las fundas y así poder limpiar todo eso, porque lo dicen en esos programas que dan de solidaridad y ahí hablan de que tenemos que tener el patio limpio también.

**¿Cómo se pueden involucrar los demás, los vecinos? Ustedes mencionaron que debemos de limpiar nuestro patio y que es lo que podemos hacer para mejor nuestro hogar, pero si no hacen eso nuestros vecinos, los problemas vienen a nosotros también, porque todo está relacionado. ¿Entonces como uno puede involucrar a los vecinos, o cuales son las barreras que se presentan en los vecinos?**

-No sé si ellos no entienden que teniendo un pozo de agua ahí, ese lodo o las matas no les hacen daño. Parece que a ellos no les hacen daño, ellos se encuentran que están bien y por aquí es de verdad que no están bien y yo me la paso limpiando.

**¿Cuándo la comunidad quiere cambiar algo por ejemplo: las calles o algo, cual es el proceso para hacer eso?**

-¡Esto si es grande! Jejeje. Ellos lo primero que hacen es ir a la junta de vecinos y hasta hacen su huelga, a ver si logran algo.

-E ir al ayuntamiento.

-Sí, el presidente de la junta de vecinos es quien presenta la queja, él va allá y nos dan una cita.

-Cuando comenzaron arreglar la calle, cuando ya ellos estaban dispuestos arreglarla y no sé qué paso, el síndico dijo: que como nosotros hicimos piquete (huelgas o reclamos) él no la iba arreglar y no la arregló. Sin embargo aquí fue donde se le dio mucho apoyo.

**Entonces a veces es difícil si no hay mucha confianza en este proceso, por ejemplo: cuales son las cosas que nosotros podemos hacer como vecinas y miembros de la comunidad para hacer algo de beneficio?**

-Hacer un operativo o hacer algo, pero es como dijo ella: tenemos que ir a la junta o donde la presidenta de la junta de vecino y decirle: mira fulana, yo quiero que para el jueves hagamos una reunión para hablarle a los vecinos de un operativo para que se acerquen a nosotros y que vengan y ayuden hacer limpieza, aquí mayormente se pone cuando está llegando la Navidad a veces todas la personas se poner a limpiar el patio y a decorar exacto hasta la basura la pagan para que se la boten.

**Y ¿cómo fue la experiencia de eso?**

-Fue muy bien. Casualmente hubo un tiempo que decoraron y había matas que eran muy peligrosas y la mocharon y el ciclón llegó y si no hubieran podado iba a ver muchas casas que se iban a desbaratar.

**Y pensando en la salud de los niños, en sus opiniones, ¿cuáles son algunas cosas más importantes para cambiar algo o mejorar algo para poder mejorar algunos de eso problemas de salud?**

-Tener más cuidado con ellos, yo la mía la dejo que juegue un chin pero cuando le digo: mire, quédese sentada ahí, ella se incomoda y la siento y le digo: mira, mami, y le doy un jueguito, le compro una galleta o algo, y ese día pasa hasta que llega la hora de dormir, la duermo y la acuesto y como en dos hora se para y le digo: déjame bañarte que ya está cayendo la tarde y ella viene calladita, yo la estoy criando para que ella se mantenga limpia, yo me acuerdo que los hijos mío cuando estaban pequeños ellos le decían a los demás: no te me pegue que me vas a ensuciar y nunca ellos se me pusieron así. Yo vivía en un batey, y esos niños se mantenían limpiecitos, y cuando yo lo iba a bañar, le ponía su ropa limpia y su mediecitas y siempre estaban lindos porque hay que cuidarlos.

**¿Y que piensan ustedes?**

-Lo mismo que ella dijo.

-Es mejor orientar en cosas que te ayudaran en tu salud.

**¿Creen que hay falta de conocimiento?**

-Concientización.

**¿Concientización del medio ambiente o cómo está relacionada la salud o la gente saben eso o que piensan?**

-Bueno yo creo que sí que ellos lo saben pero no lo hacen.

-Saben pero no lo hacen.

**Y ¿porque no lo hacen?**

-Por en ejemplo, hacemos una reunión ahi mismo o un grupo de apoyo y se le orienta acerca de los mosquitos la basura, van a durar esta semana echando la basura en funda y limpiando todo, pero en dos o tres meses ya vuelve. Uno tiene que estar hay constante.

-Se le olvidan.

-Exactamente, son personas que saben las cosas y no la hacen.

-En el barrio hay mucha basura porque las matas están botando las hojas y hay mucha basura y se llena de hojas. Yo misma barro todos los días para no ver toda esa basura vieja.

**Mencionaron la junta de vecinos como personas que viven dentro de la comunidad, el síndico, y el ayuntamiento. ¿Cuáles son las otras personas de la comunidad o en Consuelo que puedan ayudar o ser una parte de apoyo o de ayuda? ¿Organizaciones o grupos (como las escuelas) que si estén involucrada eso sería bueno?**

-Aquí no se ve mucho de eso.

**¿Cómo?**

-Que aquí no se usa mucho eso, que la escuela ayude a la comunidad.

**¿No se involucran mucho?**

-Aquí lo más que se puede es que un día venga salud pública.

-Si, como el día que vino a hacer operativos, cuando había mucho dengue. Que habían unos cuantos niños con dengue.

-Y vino salud pública y dieron una charla sobre el dengue y dijeron que iban a venir a untarle cloro a los tanques y a dar cloro y todo eso. Pero nada más vinieron a los sitios que había dengue solamente. Yfumigaron.

**¿Las charlas ayudan o no ayudan a la gente?**

-Es como yo te dije ahorita, que dan la charla hoy y solo duran una semana o dos haciéndolo pero después, no.

-El que esta consiente de lo que hay sigue echando su basura aparte, pero el que no.

-Si--los otros no.

**Entonces a veces si hay charlas con más frecuencia, osea que después de tres meses dicen vamos a hacer un pequeño recordatorio….**

-Si vamos hacer un operativo pero la mayoría no va.

-A veces no vienen y salen corriendo.

-Me gustaría que siempre si tiene la oportunidad lo sigan haciendo, a mí me gusta escuchar porque no tuve la oportunidad de estudiar o yo quería estudiar. Pero a mí me gusta mucho estudiar, me gusta mucho leer, y me gusta siempre investigar.

**Parece que las charlas y operativos han funcionado aquí pero por poco tiempo, y a veces hay personas que van a la junta de vecinos y a vece puede lograr algo pero a veces no.**

-A nosotros viene una supervisora del programa solidaridad, y ella siempre habla de eso--los patios limpios, los niños al médico, que no dejen basura amontonada que eso daña el aire y que la echen en fundas. Ella siempre mete ese tema. Habla también de que tenemos que ir al médico hacernos un papanicolau, un examen de mama, y todo eso.

**Ah si, por prevención.**

-Sí, exactamente.

**Y si con las charlas enfocan la salud de los niños y problemas, ¿la gente no le hacen más caso o no importa o a lo mejor lo mismo ayuda por un rato?**

-Es así, mientras él ni no está enfermo lo estamos cuidando y desde que está mejor le dan banda como dicen los tigueres, jejeje. Es así.

**Entonces, ¿cuáles son algunas maneras de específicas para dar ánimo a la comunidad para que piensen en estas cosas?**

-Bueno que se vean afectado como la chinkungunya cuando la gente creía que iba a acabar con todo el mundo, todo el mundo estaba alerta.

**Ah, que cuando hay un problema es que se ponen así.**

-Mira, yo siempre en la calle, siempre yo ante de darme la chikungunya y agarraba ese patio desde una casa hasta casi cuatro casas hasta abajo así porque siempre me gusta ver la calle bonita y paso el día recogiendo basura en el patio y todo. A veces le digo a mi hermana: tiene que ayudarme que yo tengo yo sola no puedo está recogiéndola la niña se me está poniendo jodona, jodona. Ahora, lo que quiere es coger para la calle y tengo yo que estar pendiente de ella y yo lo que vivo es que es de mi negocito osea de hacer mis cosas, entonces yo digo a mi hermana: agárrame la niña ahí porque tengo que limpiar y tengo que hacer esto. Y yo le digo a ellas que tienen que involucrarse el en la madre porque le ayuda mucho pero los jóvenes de allá se descuidan mucho y todo. Me lo dejan a mi sola yo le digo el patio es muy grande, mire mi hermano botó todo ahí atrás pero ahora toda esa basura está ahí atrás. Ahora tenemos nosotros que limpiar todo esos palos para recoger todas esas hojas y echar toda esa basura en funda grande y en saco.

**¿Ustedes creen que sería interés entre adolescentes como tener un grupo como “un equipo verde” o algo asi--un equipo de adolescentes que viven en el barrio y que pueden hacer cosas para ayudar a los vecinos?**

-Ellos si uno le dice ellos ayudan, mira ven para que mañana me ayuden a decorar todo esto, ah si no hay problema, algunos me dicen: tía si yo la ayudo y le digo ya saben que mañana lo voy a llamar para que me ayuden y así y los hijos mío me ayudan.

**¿Qué piensan ustedes?**

-Algunos ayudan por algo, y le digo mañana te voy a esperar para que me puedes hacer eso ahí.

**A veces algunas comunidades han tenido éxito con un grupo de jóvenes que pueden tener su energía y le gustan las cosas así. Bueno, entonces hay algo más que no hemos discutido que ustedes quieran decir? Hay más opiniones, o más preocupaciones o pensamientos sobre cualquier cosa que no hemos hablado?**

-También algo que no hemos mencionado que es el tendido eléctrico, eso está acabando. Los alambres no sirven--se queman y se parten principalmente en la otra calle por donde yo vivo. Los alambres se parten diario, en un día hasta tres veces se caen al suelo donde hay tantos niños. Eso alambre no sirven no aguantan la luz.

**¿Y afecta la luz?**

-Sí.

-Y es peligroso para los niños.

-Sí, los otros dia compre siete libra de cereza y una guayabas y la herví y la tenía ahi para licuarla. Duró tres días sin luz por un problema que hubo en general, pero después vino alguien voluntario porque entre veces aparecen voluntarios porque no tiene en la casa porque están afectadas también con el apagón. Hay gente que saben bregar con eso y vienen y se suben pero si no se animan--nos quedamos a lo oscuro.

**Algunos de ustedes piensan algo de la luz, se va con mucha frecuencia?**

-Si se va mucho. Mucho.

**Y ¿cuál es el proceso cuando está dañada, arreglarla, pero cuando viene un voluntario eso es bueno, pero a quien tienen que reforzar?**

-Uno va al PRA y va la presidenta o una de las personas que están pagando con tarjeta y van allá y dicen mire hay un problema en [este barrio].

**¿Y van?**

-Hay veces que ellos no lo pueden resolver y lo reportan a San Pedro y de San Pedro vienen.

**Y sin luz eso afecta casi toda la vida.**

-Eso produce gastos porque hay que buscar gas y velas porque uno no puede estar en esa oscuridad.

-Y si uno tiene cualquier cosa en la nevera ya se le daña a uno.

**Eso es un problema que tiene que ver con el medio ambiente. Hay una parte de la comunidad que quiere hacer algo sobre eso o sobre la electricidad porque cada uno cuando tiene problema tiene que buscar la manera de solucionarlos?**

-Estamos planeando un grupo ir al PRA a ver si ellos pueden venir a resolver ese problema. Alguien dice que ellos no dan alambres, pero lo que necesitamos es que vengan a arreglar el tendido eléctrico porque eso no está bien, eso afecta y a cualquiera le puede caer arriba.

-Tal vez no lo mate pero lo va a dejar bien achocado (maltratado).

**Pues gracias por decir eso, algo más? Muchas gracias otra vez por su participación.**
