# Supplementary material for: Talking trash: Perspectives on community environmental health in the Dominican Republic
Source: PLoS One. 2021 Mar 29;16(3):e0248843. doi: 10.1371/journal.pone.0248843 (PMC8007031; doi:10.1371/journal.pone.0248843)
Supplement: S7 File — (DOCX) [file pone.0248843.s007.docx]

***(Introducción)…***

**…Ustedes compartieron sus opiniones sobre problemas de salud más comunes… (*Explicación*)**

1. **La fiebre**
2. **La gripe**
3. **Vómitos/Diarrea**
4. **Parásitos/Amebas**
5. **Dengue/Chikungunya**
6. **Infecciones de la piel**

**Vamos a discutir en grupo.**

**¿Qué piensan ustedes de este listado—falta algo?**

-Creo que no.

**¿Cuale es el problema más común, a lo mejor no sea el más grave pero lo más común?**

-Los hongos que no lo veo ahí--o es lo mismo que infecciones de la piel?

**Infecciones, ok. Hablanos más sobres los hongos.**

-Aquí yo veo muchos niños con hongos, abundan mucho, mucho. Muy común en la cabeza.

**¿Cuáles son la que pueden causar muchos problemas pero no afecta tanto a los niños--solo que se complica? Por ejemplo hay niños que presentan diarrea y la diarrea a veces sucede solo porque la gran mayoría son virales, pero hay muchos niños que no que incluso esa diarrea lo puede llevar hasta a un quirófano.**

-A ellos se dan—precisamente la niña mía tiene pero ella esta normal, no se le complica ni nada ella está bien normal.

-Pero a veces le da una gripe y después llegan a una neumonía. Claro aunque se le vea normal, pero dizque se le quita y sigue hay.

-Lo más mal es la gripe por aquí, y afecta más a la sociedad.

-Sí. Y han ingresado aquí varios niños por gripe.

-Cuando la hermanita mía se congestiona, es que mi mama la saca como dos y tres veces pero eso depende. Ella se congestiona mucho.

**¿Hay muchos niños aquí que se aprietan?**

-No se te decir pero mi hermanita se aprieta mucho.

**Ok. ¿Había mucho dengue aquí?**

-Si hay mucho sí.

**Si ¿hay muchos casos de chinkungunya?**

-Uff, chikungunya acabó con la gente. A mí me dio dos veces eso.

-A mí me dio y le dio a todos en la casa.

**¿Y porque había tanta chikungunya aquí? ¿Qué opinan ustedes?**

-Bueno. Eso era algo como un virus. Es una epidemia porque eso llegaba a la casa y arrasaba con todos.

-Eso fue algún virus porque el aire contaminado, porque eso es por lo que afecto tanto, usted me entiende. Dicen que dizque es mosquito, y si fueras eso yo estuviera muerto porque aquí si hay muchos mosquitos. Aquí hay muchos mosquitos.

**Ok… ¿Aquí hay muchos mosquitos--más que en otras partes de Consuelo?**

-En todos los sitios hay mosquitos.

-Bueno, para mí en este barrio es que más mosquito hay porque este es el barrio como es un sitio perdido así, usted me entiende. Abandonados por todos. Es verdad sí.

-Jejeje sí.

-No, pero es verdad.

-No más o menos, excúseme de lo que estamos hablando, no es cosa de políticos. Es como dicen si ni hay unión en un sitio donde uno vive, más o menos con la limpieza, entonces usted sabe que ese sitio va a ver muchos mosquitos y va a ver de todo un poco, entonces por eso es que se habla de la limpieza, usted me entiende.

**Que la limpieza tiene que ver con la cantidad de mosquitos**?

-Sí.

**¿Hay otras opiniones?**

-Bueno, la higiene es una de la cosas importante porque por vía de la higiene es que se evita muchas enfermedades y contaminaciones y eso es una de las primeras cosas que hay que llevar pendiente.

**Ok, entonces hablando de eso vamos a seguir entonces, ustedes también mencionaron algunos problemas en el medio ambiente y que causan problemas en la salud:**

1. **Mosquitos**
2. **Aguas sucias/Pozos de agua**
3. **Condición de la calle**
4. **Basura**
5. **Humo de quema de basura**
6. **La luz y los alambres (que se caen y pueden causar problemas con los niños cuando están jugando)**

**Quiero escuchar un poco más--es una oportunidad para compartir que ven ustedes en la comunidad.**

-Los alambres porque yo vivo por allá, y hay un alambre que cruza por mi casa y ese alambre a cada rato se cae, y son de aluminio y hay riesgo que se caiga y mate a cualquiera ahí.

-Lo cable de alta tensión.

-Sí.

-De eso alambrito fino de aluminio.

-Pero que tienen un alto voltaje porque por eso es que se parte.

-Son como así pero de aluminio.

-Y están medio a medio.

**¿Eso pasa en muchos sitios?**

- Sí. En gran parte del barrio porque por allí ni anda pasando eso.

-Eso se ve más de aquel lado.

-No, en aquel lado por lo menos está bien, tuve su cable pero por este lados que está más mal.

-Si hay se caen a cada rato pero eso no pasan por arriba de la casa.

-Y esos alambres también están muy bajito.

-Que no eso es que lo palo no sirven.

-Tiene que hablar e ir al ayuntamiento y juntarse la gente que viven cerca y lo sacan de que lo sacan lo sacan.

-Si porque hay unos por allá que parece que han hecho un grupo, pero es que la gente de allá no quieren eso.

-De cualquier brisita que hace se caen, hace días el niño mío estaba parado al frente de la calle. Yo iba cruzando con él y él se paró, y ese alambre nada más se partió, yo digo que si se parte más largo mi hijo no tuviera vivo, tuviera siete metros bajo tierra.

-Y se caen también lo de la calle de aquí porque cuando uno sale de aquí del otro barrio al lado, tu sabes donde se mató el muchacho--te acuerda ahí, ese se cayó el otro día también.

-Y la quema de basura también aquí frecuenta bastante, sí, porque cuando yo estaba embarazada y prendieron una basura eran como las once de la mañana y cuando la prendieron yo tuve que salir y dejar la casa porque ya no podía respirar y me estaba asfixiando, porque parece que estaban quemando era un humo toxico y me estaba haciendo mucho daño. Por allá queman mucho también.

**Eso le ha pasado a otras personas?**

-[Ella], la de al frente, ella se aprieta y allá a plena doce del día es un palo, cuando estaban construyendo allá mi mama fue allá y le dijo que esa niña se aprieta y en esos días esta misma estaba mala también y me mando a decir que le dijera yo y cogí una cubeta de agua y se la eche y le dije y voy para el cuartel ahora mismo y vuélvala a prender para que usted vea y más nunca lo prendió.

-Aquí hay que quitarlo porque él dice una cosa y lo tienen de relajo y el como presidente de vecinos que es y lamentablemente le pasan por arriba y hacen lo que quieren. Eso es una realidad lo que tu dice no lo respetan.

-Por eso yo me hayo que el barrio esta así como esta porque es que nadie lo apoya, él dice algo y la gente hacen lo de ellos mismos a su manera eso si es verdad.

-Sí. No hay unión.

**¿Y porque piensan ustedes que queman basura?**

-Queman basura porque muchas veces los camiones no la recogen.

-Eso si es verdad.

-Si porque hay veces que duran hasta tres semanas sin venir a recoger la basura, y vienen cuando ellos quieren.

-Pero si tenemos conciencia, porque yo a veces he acumulado hasta diez sacos de basura en mi casa porque no la puedo quemar, porque sé que me hace daño a mí y a los demás, y eso es lo que pasa—que a veces no tenemos conciencia.

-Tú sabes que la otra noche, fui con dos sacos nada más de pampers, tu sabes que este niño usa pampers para dormir y cuando duro tres semansa y después dizque que vino y se dañó y no volvió yo tuve que ir a botar esos pampers lejísimo porque eso es lo que pasa. La abuela mía tuvo que ir a botar los pamper en un saco.

**Entonces, cuando el camión no viene a recoger la basura ¿Cuáles son las opciones? Quemarla, andar muy lejos a botarla en un sitio…qué más?**

-Andar muy lejos a botarla porque nosotros particularmente no la andamos quemando porque eso afecta en verdad por donde yo vivía. Pero a veces tenemos que salir alguno para allá porque uno no la va a acumular como dice ella hasta que ellos quieran.

-Sí, acumularla hasta que ellos quieran, pero también hay personas que no tienen ese espacio de acumular porque yo por lo menos la pongo por allá atrás y no me molesta tanto el mal olor y eso porque esa basura y las moscas eso molesta adentro de la casa, y más si tienen varios días esa basura y cayéndole agua hay, si es verdad.

**¿El basurero está cerca?**

-Bueno, no cerca que digamos pero…

**¿Más cerca del otro barrio al lado?**

-No, de aquí queda más cerca.

-Si usted baja en esa bajadita ahí, y esta el basurero ahí mismo.

**Ok ¿Y qué le pasa a la basura allá?**

-La queman.

**¿Y el humo de eso llega hasta aquí?**

-Si, eso le hace daño.

-Aquí no llega. Del basurero no llega aquí.

-Como más o menos tú sabes que no llega.

-Que no llega. Es que no llega.

-Aquí mismo de allá no llega. Allá no llega tampoco.

-Aunque a ese basurero deberían de buscarle otra solución porque este barrio está muy grande y ese basurero está cerca, cerca, y todas esas moscas salen de allí y vienen para el barrio.

-Claro, y todo eso es contaminación porque de un punto de vista y [el barrio al lado] casi esta encima del basurero. Eso si es verdad porque hay casas hay mismo.

-Sí, sí. Se ha llenado de casi todo eso.

**¿Y qué sería lo mejor solución en su opinión? ¿Que se podría cambiar?**

-Bueno, yo pondría el basurero más lejos porque eso es un barrio ya y por vía de ese basurero al estar tan cerca de esa comunidad las moscas viven más en los hogares que en el mismo basurero. Porque ya está muy cerca lo que hay que hacer es como un traslado a ese basurero y eliminarlo de ahí totalmente y ponerlo más lejos—y cuando lo pongan más lejos tratar de que la gente no empiece a invadir más terreno porque eso es lo que pasa también.

-Sí.

**¿Y si no se puede mudar el basurero hay otras cosas que se pueden hacer? Como nosotros que vivimos aquí por ejemplo: para reducir la cantidad de basura?**

-Reducir la cantidad de basura.

**¿Cómo pueden? Porque es muy difícil osea en muchas partes del mundo es lo mismo que no hay muchas alternativas y hay mucha basura, somos humanos y producimos mucha basura.**

-Innecesariamente.

**Pero a veces no se pueden cambiar esas cosas…bueno a lo mejor pueden mudar el basurero pero si hay otras cosas que podemos hacer en la comunidad, como unirse y cambiar algo para reducir lo que está en el basurero o que no quemen cosas toxicas. ¿Seria de interés para las personas que viven aquí aprender reciclar o por ejemplo: coleccionar cosas de vidrio, cosas de metal y echar en un lugar para llevar a Santo Domingo, a un sitio que cambia a otra cosa en vez de llegar al basurero? ¿Tendrían interés en eso o que piensan?**

-Eso lo han hecho aquí…Separar la basura.

-Pero imagínate tu yo no me voy a poner en mi casa a entre sacar la basura y ciertas cosas y ellos lo hacen cuando están en el vertedero me entiende.

-Pero podemos ir poniéndolo en funda, esta funda es para los platicos, esta funda para la comida.

-Ah ponerla aparte todo aparte, ok. Si lo que es de vidrio de vidrio y lo que es de plástico de plástico, cada cosa en su lugar.

**Y lo que pasa es que cuando está separado así en vez de botar todo en el basurero o donde la queman ya está separado y llega menos cantidad al basurero y el resto lo llevan a otro sitio como una fábrica que hay que hacen otras cosas con los de vidrio, con el metal, eso es algo que en otras parte del mundo es lo más ecológico en el sentido de que no tenga tanta basura que la quemen o que la boten.**

-Bueno pero esta difícil para hacerlo, difícil.

-Aquí no hay una empresa que se encargue, porque la mayoría lo que compra es hierro y plástico, pero hay una señora que vivía por ahí atrás que ella recogía plástico.

-Y aquí bajan varios camiones a recoger todos los platicos y potes.

-Si pero eso no viene por la educación porque hay gente que hacen su negocio solos. Porque a veces se puede ganar dinero.

-Sí.

**Porque se vende eso más o menos y algunas comunidades han empezado proyectos asi y si coleccionan una cantidad grande y se vende a este negocio que lo lleva a otro sitio se puede tener un chin de dinero. Entonces vamos a hablar un poco de la condición de la calle ¿Que ustedes piensan de eso? ¿Y porque es un problema?**

-Bueno, por aquí mismo es un problema si, porque cuando llueve ahí adelante por donde vive esta muchacha no se puede cruzar por el lodo, el charco de agua, y se pone muy feo eso por ahí.

-Por ahí por mi casa cuando llueve el agua mira porque por ahí no hay zanja. Usted vio la calle y cuando cae toda esa agua se mete hasta adentro de la casa.

-Sí.

-Si porque el otro cuando yo pase por ahí, ahí está jaraneando.

**-¿Entonces eso tiene que ver también con mosquitos cuando ahí charcos de agua?**

-Es que imagínese al haber tanto monte cuando llueve.

**Entonces yo creo ya que hemos mencionado algunas cosas, pero realmente quiero escuchar como pensando en el futuro ya que estamos de problemas que vemos en nuestra comunidad que tiene que ver con la salud y tiene que ver con el medio ambiente, es muy bueno hablar de esas cosas pero lo mejor es pensar como un grupo de vecinos aquí ¿Qué podemos hacer realmente para cambiar cosas o mejorar cosas? ¿Cuáles son algunas de las barreras a unir y cambiar? ¿Cómo puede trabajar juntos la comunidad para mejorar el medio ambiente de este barrio y hacerse mejor para la salud de los niños?**

-Bueno, yo diría uniéndose con el presidente de la junta de vecinos porque ese es otra cosa que aquí como el presidente de la junta de vecinos cuando el barrio tiene que ir solo a tocar puertas y bien se dice presidente de la junta: que es una comunidad no una sola persona. Entonces que se debe hacer, hacer una comisión y cuando se vaya a tocar puerta que no vaya él solo, porque a él solo no lo van a escuchar, si ellos van un grupo de diez o quince personas así si le pueden hacer caso, porque ven la necesidades por la que él está pidiendo y le pueden resolver más rápido.

**-Pero es el presidente de la junta de vecino de aquí donde ustedes viven.**

-Si el de aquí.

-Yo trabajo con la junta de vecino yo soy la secretaria.

-Ah esta en eso.

-Sí.

**-Y usted porque no se ha unido con el presidente de la junta de vecinos?**

-Yo, yo no tengo tiempo para eso.

-No yo como le digo yo cualquier cosa siempre yo estoy con él y más que yo no paro aquí mucho, yo no paro aquí.

-El mismo dice vamos a preparar una… mira para ese mismo problema de la luz ultimamente habían unos apagones en la noche y yo misma le dije a el vamos a preparar a ver quiénes se animan y vamos hacer una comisión para y vamos a ir a ver si se resuelve, porque aquí últimamente te estaban atracando a las siete de la noche y aquí nadie se animó a eso porque todo el mundo tiene compromiso y nadie podía sacar una media hora y nadie quiso hacer esa gestión solo quieren que él lo haga solo y el solo no puede, porque el solo va a tocar puertas y no le van a hacer caso.

**¿Ustedes creen que hay suficiente conocimiento de problemas con la salud y la relación al medio ambiente o que no saben la gente?**

-Ellas si saben. Claro que saben. Si saben.

**¿Pero entonces cual es el problema? Bueno ya hemos mencionado muchas cosas ustedes están compartiendo de su comunidad y de lo que ustedes observan y algunas ideas a lo mejor para mejorar, ustedes creen que hay algo que se puede apoyar como ayudar pensando en la salud de los niños y la medio ambiente ¿Hay algo más que puede hacer?**

-(silencio)

-Hablen, hablen porque ustedes se quedan callado.

**Es que este proyecto el objetivo es pensar en lo que podemos hacer como una organización que trabaja con la comunidad y que nuestra meta es mejorar la salud de los niños y pensando en el medio ambiente y que ustedes mencionaron que hay varios problemas que afecta la salud de** **los niños, entonces vamos a escuchar a todos sus ideas y las ideas de los otros barrios para ver lo que tiene sentido para seguir adelante y enfocarnos.**

-A ver si pueden ayudarlos.

**Si exacto, pero pensando en casa como más orientación, o concientizarlos a los vecinos, o trabajar con un grupo de personas que están interesados en hacer algo o no sé, estamos abiertas a ideas entonces queremos escuchar de ustedes, no sé si piensan en otra cosa ahora.**

-Trabajar con grupos que estén interesados y que sepa que puede sacar ese tiempo para cuando ese momento llegue que esté disponible para eso, porque en nada estamos que sí, que si pero en su casa saben que no va asumir su responsabilidad como tú dice, pero que si no va hacer responsable que mejor diga que no y no lo acepten.

-Por lo que yo he visto sería bueno como que se junten con lo que es la junta de vecinos y así con la junta de vecinos directamente trabajar en la comunidad ¿en cuáles? En diferentes proyectos como concientización de la separación de la basura porque eso no es difícil porque todos sabemos lo que es plástico, lo que es metal y lo que es vidrio, todos sabemos lo que es eso entonces solamente es yo sé que lo que nos falta es orientación sobre cómo hacerlo y motivación sobre todo y eso sería una propuesta que lo harás propiamente como la junta de vecinos, y con la junta de vecino tratar lo que es la comunidad y quienes son lo que conforman la junta de vecinos son la misma comunidad, entonces asi es más fácil llegar a la comunidad a traves es la junta de vecinos.

-El hombre que compra plástico tiene su número de teléfono.

**Si exactamente porque hay un que pasa por Consuelo y viene y colecciona plástico y otras cosas, y tengo una reunión mañana con otra persona porque en otra clínica donde trabajos vamos a empezar con un programa de reciclaje entonces si hay logro de nuestra parte a lo mejor podemos hacerlo aquí, porque es una manera de reducir la cantidad de basura y si reducimos la cantidad de basura, reducimos la cantidad de quema de basura, y reducimos la cantidad de estar acumulada en un patio o molestando de bajo de la lluvia. Pero ustedes han mencionado que a veces nos sentemos hablar con un vecino y no hacen caso o no tienen interés o eso es un problema?**

-Que no hay unión, igual que la junta de vecinos se le propone esto y mucha veces “ay no..es difícil yo no puedo.” Que prácticamente no hay unión y no se apoya la junta de vecinos.

-Es que todo el mundo aquí resuelve su problema como puede.

-Jejeje.

-Es la realidad, un ejemplo que se caiga un alambre en frente de mi casa pasan alambre y que se caiga uno los únicos que van a aportar somos mi vecinos y yo.

-Es verdad. Por eso es que el barrio esta así como esta.

-Eso es una realidad, es como dice [ella]: lo alambre de allá de donde la viuda cuantas veces no se han caído y la gente salen, la gente salen, la gente no salen. Yo salgo porque mi luz viene de por ahí y voy al pra y a veces la gente vienen y uno tiene que pagar para arreglarla.

-Ese es el problema que se daña y dizque la arreglan pero la ponen supuesta como para que vuelva y se caiga, entonces ello no hacen nada.

-A veces si uno no busca doscientos o trescientos pesos no la arreglan.

-No es lo mismo--así yo tenía ese problema que lo mío se caían a cada rato y yo semanalmente tenía que pagar para que me la pegaran.

-Allá si se cayó uno busca trescientos y no la arreglan y nadie sale.

-Venga fulana cincuenta pesos para la luz, no yo no tengo, ven después, yo no tengo ahora.

-Esos problemas todo el tiempo van a suceder hasta que no cambien esos alambres.

-Es igual como con los tubos del agua aquí, que aquí todo el mundo dice que va a comprar tubo y nadie lo compra y todo estamos yendo para [el otro barrio al lado] a buscar el agua. En mi casa no han puesto el agua, pusieron agua en todos los lados para allá. Pero ninguna de esas casas para allá abajo tienen agua, ninguna. Es para [el barrio al lado] que van.

-Pero es que ya el esfuerzo él lo hizo porque el trajo la tubería pero fue el solo porque el solo es el que toca puerta y le donaron sesenta tubo y aun así él tuvo que compra como diez tubo de su bolsillo.

-Eso si es verdad que él lo compro y fue él solo porque esa agua llegó aquí porque yo hice la carta, se le llevó al síndico y el síndico fue que aportó los tubos y aun así quieren como que se la lleven directamente a su casa y a domicilio.

-Eso si es verdad.

-Pero ya que tiene la tubería central y entonces ya eso depende de cada persona conectarse, osea en esta calle a diez personas que ajunten su dinero.

-Y son tan desunidos que mejor prefieren gastar tres mil [pesos] no aportar trescientos en la casa si no tres mil en par poner su tubería él solo y no quiere que nadie se conecte de ahí entonces.

-Es verdad mira allá en la calle donde yo vivo eso es alquilado donde yo vivo y la única casa que no tiene agua por ahí es esa, hicieron una recolecta en ese callejón y la mujer de la casa dijo que iba a hablar con su marido, pero el marido no está aquí que es el dueño de la casa--el esta allá afuera, y yo le dije pero tú eres que vienes a cobrar casa entonces mi hermana vino la abuela de ella que vino hace quince días y yo le dije compra lo tubo y descuéntaselo de la casa y ella dijo que no y nosotros compramos una manguera y la ponemos del patio de mi mama que vive atrás y yo le dije no compre tubo para después dejarle agua a ellos y ellos no quieren aportar mejor vamos a buscar otra casa porque esto lo estamos pagando y ella muy nada más dizque si, vino ahí ayer dizque a medir la tubería de adelante dizque si la vamos a poner y todavía no ha aparecido. Mi hermana le iba a pagar tres meses de casa y yo le dije que no págale un solo no le pague nada.

**-Ustedes piensan que una comunidad que depende de la junta de vecino?**

-No, directamente somos nosotros mismos.

-Si es que la junta de vecinos se enfoca en que se apoderen de su comunidad, aunque colabore la junta de vecino pero si la junta de vecino no quiere. Un ejemplo el barrio no se va a caer porque la junta de vecino.

-Mejor pelean y se pegan.

-Jejeje.

-A la mala es verdad sí.

-Y los demás dicen que somos malos, pero para ahí atrás ese agua ni jugando, eso es por donde vive la mama de ella ni lava puede entonces es un lio con esa gente para ahí atrás porque no saben convivir.

-En esa calle donde vive tu mama no puede caer ni una gótica de agua ahí porque ya es un lio.

-Osea quiero a dar a entender que ella se pone a que nadie vaya a la puerta de nadie quiere hacer la cosa como ella dice solo que no es dizque con junta de vecinos y no es que lo demás no quieran compartir.

-Hay una callecita y usted sabe lo que hizo esa mujer…Jejeje.

-Es que la gente no tienen costumbre entonces dicen que es que no quieren compartir.

-Mire ella cogió una línea de piedra así y atajo la calle. Ahí no puede jugar ni un muchachito. Y uno por evitar problema porque vivimos ahí. Y ella lo hace pero si hubiese visto más vecinos pudiéramos unirnos e ir allí al ayuntamiento. Ella le puso la piedra así, la mitad de la calle cogió.

-Y los muchachos no pueden tirar ni una hojita en el suelo. Recojan eso y le comienza a decir cosa que los muchachos hasta se van gritando.

-Eso es lo que pasa para ahí atrás tienen un mal vivir y no saben compartir para nada, no quieren compartir, no es que no tengan educación y no es que uno no quiera hacer las cosas, es que no quieren juntarse con una junta de vecino. Y más ahí todo el mundo le tiene miedo a ella y ciertas personas más porque lo que le dan es boche a uno cuando uno va al frente de su casa a decirle esto, a no, si se cae el alambre a veces dos y tres casa no tienen luz, a “no vecina mire puede ayudar con cualquier casa,” a no yo tengo luz en mi casa arréglense como ustedes puedan, y ya uno lo que tiene es temor de ir entonces imagínese usted.

-**Pero la junta de vecino va a aquel lado?**

-Claro pero él le tiene miedo a esa mujer.

-Yo no visito.

-Él ha ido pero él le tiene miedo. El si él ha ido pero como te digo es una persona que no le gusta ofender a nadie y él es sencillo pero yo no yo ni siquiera sé quién es.

-A veces van los cristianos a invitarla para la iglesia y cosa y le cierra la puerta. Los otros días en la invitación de allá arriba dizque yo no le he dicho a ningún cristiano de que se yo que, que venga a mi casa ni a invítame a la iglesia.

-Ella es así y lo demás lo que le hablan feo a uno entonces para no tener problema con ellos, mejor resuelve como se puede, si ella no tiene amen si el otro etc. usted entiende lo que le digo, o me entendieron, no sé si me entendieron.

-Jejeje. Yo creo que esa mujer vive la vida desesperada.

-Jejeje. Ella no es sola son varias personas.

-Esa es peor.

-A ella la están haciendo pasar por loca donde el fiscal, y ella no es loca nada ella sabe lo que hace, pero le tiene miedo, usted ve.

**¿Algo más que quieran compartir?**

-Yo no, ya.

**Estamos aquí para escuchar esas opiniones porque ustedes son lo que viven aquí y estamos aprendiendo mucho. Entonces hay algo más que no hemos discutido?**

-No ya. Está bien.

**Pues muchísimas gracias por su participación, sus opiniones realmente son muy importante y valiosas y gracias por compartir con honestidad, y gracias por su tiempo.**
