# Supplementary material for: Talking trash: Perspectives on community environmental health in the Dominican Republic
Source: PLoS One. 2021 Mar 29;16(3):e0248843. doi: 10.1371/journal.pone.0248843 (PMC8007031; doi:10.1371/journal.pone.0248843)
Supplement: S8 File — (DOCX) [file pone.0248843.s008.docx]

***(Introducción)…***

**…Ustedes compartieron sus opiniones sobre problemas de salud más comunes… (*Explicación*)**

1. **La gripe**
2. **La fiebre**
3. **Vómitos/Diarrea**
4. **Amebas/Parasitos**
5. **Chikungunya**
6. **Infecciones de la piel**
7. **Tos**

**Vamos a discutir en grupo.**

**¿Que ustedes piensan? Oigo muchos sí.**

-Estos son lo que más ataca a los niños en [el barrio].

-Para mí es la gripe y la fiebre.

-La gripe y la fiebre.

-La diarrea y los vómitos.

-Y la chinkungunya también dio para matar a los niños, usted se acuerda.

**¿Porque es muy común la gripe, según lo que ustedes piensan de [este barrio]. Porque es tán común?**

-La gripe es común porque hay mucho polvo y mucha contaminación porque ahí pasan muchos vehículos con humo contaminante de los aparatos.

-Y cuando llueve también porque no tenemos desagüe en la carretera y más en estos días de los aguaceros y el agua nos daba a la rodilla, todo mojado eso era un desorden increíble. Y la calle llena de agua también y no había por donde salir. Muchos moqsuitos. Pero acuérdate también la chinkungunya estaba atacando fuertemente era que teníamos ese aguacero adentro de la casa y todo eso es porque no tenemos desagua, cuando llueve que se nos queda el agua aposada y dura muchísimos días el agua aposada porque no tiene por donde irse por la falta de desagua, todo eso lo que trae es contaminación a los niños, a los grandes, a todos.

**¿Y de dónde o porque hay tantos vómitos y diarrea entre los niños? Qué opinan ustedes?**

-A ella casi nunca le anda dando eso. Ella es solo le da gripe fiebre pero no eso de diarrea.

-Bueno al mío no le anda dando vomito ni diarrea pero me le dio ameba en estos días. Allá es la gripe que ataca mucho. Le dio amebas e infección en la orina.

**¿Porque tenemos tantas ameba?**

-También a los niños le dan los vómitos y diarrea cuando ellos recogen algo del suelo y tienen la mano sucia. La misma contaminación. Y vienen y se la mete en la boca y por eso es que le da vómito y diarrea.

**¿Otras ideas? Hay muchas infecciones de la piel?**

-[Infecciones de la piel] tienen que ser por las picaduras de mosquito, cada vez que los mosquitos pican un muchacho se le hace una roncha y le da rasquiña y eso es de la picadura del mosquito.

**¿Y tenemos muchos mosquitos aquí?**

-Claro aquí hay muchos mosquitos. Mucho, mucho. Y más por ese campo de caña que esta allí.

-El agua sucia eso es lo que da muchos mosquitos.

-Sí. Y nosotros como vivimos parte atrás, para hay atrás hay campo de caña. Y basura.

-Sí.

**La basura?**

-Sí, porque hay alguna gente que tiran la basura para ahí por tu casa, el camión de la basura—hay sábados que él no vienen, entonces la gente tiran la basura para el campo de caña y eso es lo que trae más mosquitos y contaminaciones.

**¿Y alguien puede explicar cómo está la carretera y el barrio, porque ustedes mencionaron que estar cerca de la carretera y de la caña, entonces alguien puede explicar esto?**

-Nosotros principalmente quedamos y la calle esta adelante pero nosotros quedamos más pegado de atrás que de adelante.

-La caña nos queda ahí a esa esquina y nos quedaba más cerca pero hubo un fuego el año pasado y se no iban a quemar la casa y ahora la retiraron más para atrás.

-El síndico habló para que lo quitaran pero estamos más cercas. Estaba casi adentro de la casa estaban la caña.

-Así mismo, mira cuando ese fuego lo único que hicimos fue que cogimos los niños y salimos para la carretera y íbamos a perder todo. Con el fuego de la caña**,** toda la mata toda, toda se quemó.

**-Y ¿cómo se originó ese fuego?**

-Bueno a los contratistas le dio fuego para que el fuego se fuera para adentro. Hasta los que vivimos aquí adelante tuvimos que salir. Tuvimos que salir todos huyendo.

-Hace como dos meses hicieron otra también, la caña la quemaron y llamaron a los bomberos y todo el mundo tuvo que salir para afuera para la carretera porque le molestaba eso en el aire, el humo viene contaminado porque eso también es basurero y tuvimos que sacar los niños e irnos para afuera.

**¿Algunos niños en ese momento tenían dificultad para respirar, o algún problema?**

-Si porque mire, el de ella, el de ella se puso malo.

-Y la mía también, el humo venia para mi casa y tuvimos que salir para afuera.

**Gracias por compartir eso. Ya mencionaron algunas cosas y aquí está el listado de ustedes de problemas en el medio ambiente:**

1. **La basura**
2. **La quema de basura**
3. **Las aguas acumuladas, aguas sucia o aguas negras**
4. **Los mosquitos**
5. **El polvo**
6. **El agua por el sentido de que a veces no viene y no ahí agua en las llaves y no hay agua en la comunidad, y algunos mencionaron que a veces hay tuberías rotas que bota agua y eso colecciona y causa agua acumulada y eso lo preocupa algunos de ustedes y a sus vecinos**
7. **La luz, que es un problema porque a veces no viene y se va con mucha frecuencia y se hace muy difícil cocinar o hacer cualquier cosa en la casa o en la oscuridad.**
8. **Letrinas y sanitarios – que algunas personas no tienen donde ir al baño.**

**Quiero escuchar de ustedes un poco más de sus experiencias con todo eso.**

-También hay mucha gente que no tienen baño, entonces en ese campo solamente hay caña y caña y hay gente que como queman la caña vienen con ese aire y todas esa contaminaciones.

-También hay muchas letrinas rotas, entonces por eso es el mal olor y los mosquitos vienen de ahí mismo y eso también nos hace daño a nosotros ese mal olor.

**¿Las letrinas rotas, cuando llueve es un problema?**

-Si porque sube el mal olor [de las letrinas] y también se llena y el agua se riega.

**¿Qué más experiencias con letrinas o problemas con sanitarios tienen?**

-Eso afecta mucho si, entonces la gente no puede estar tranquila con ese bajo y el mal olor y eso no está afectando mucho a las personas, esas personas que no pueden estar tranquilo por el mal olor y eso es muy complicado de las letrinas.

-También cuando llueve y cae mucha agua eso a uno le molesta mucho, entonces por allá hay muchos niños y esa agua así y esos niños. Yo quisiera saber porque esa agua le hace mucho daño a los niños porque ello hay mucha agua acumulada por allá, por allá se acumula demasiada agua.

-Y hay gente también por donde vive mi tío y por donde vive ella que hay gente que agarran y tienen agua y manguera y bota toda esa agua y se acumula. O si no cuando están llenando algo la dejan así como que no hay nadie que pase por ahí, imagínate nadie se conduele de nadie.

-Por mi lado nosotros estamos bien en el pedazo de nosotros, ahí no se acumula agua y puede llover lo que llueva y el agua se va porque nosotros estamos en un alto por eso está bien.

-Allá el agua baja en seco. Allá no se acumula agua.

**Entonces algunas parte del barrio acumulan mucha agua y en otros no.**

-Allá el problema casi todo el tiempo todo el mundo tiene su baño. Allá nosotros no tenemos problemas gracias a Dios. Allá en el pedazito donde vivimos pero ellos también viven en otro lado.

-Pero es que hay gente que no dan mantenimiento ni al baño tampoco. También hay muchas gentes que no tienen letrina por allá. Y otros lo tienen pero no dentro de la casa si no afuera, porque allá mismo en mi casa el baño está afuera.

-Yo también lo tengo afuera.

**En lo que mencionaron, que no se acumula tanta agua ¿Hay menos o más mosquitos allá?**

-Los mosquitos no, porque tú ves los mosquitos ya gracias a Dios el vecino limpió toda esa área ahí tuve ya eso está limpio y no, no afectara mucho los mosquitos porque eso es lo que trae todo esos pájaros y ratones y cosas que nos están acabando a nosotros y no podemos aguantar, la ropa y todo lo dañan.

-Mucho ratones. Oye! Allá en el patio hay mucho. Hasta las chancletas se comen yo estaba hasta gritando y una algarabía.

-Eso solo lo trae la basura. Mi hija, ellos se esconden hasta abajo de la cama. El gavetero yo lo tengo vacío.

-Yo también tuve que sacar toda la ropa porque me lo tenían lleno de guayaba, de mierda de ratón y en siendo cosa que encontraran la metían toda para el gavetero.

-El mío yo lo tengo vacío en funda lavada la tengo. Mucha ropa me ha dañado a mí. Asi mismo. Todo lo que es bata piyama todo me lo dañaron.

-Yo no puedo ponerle veneno porque entonces los muchachos…

-Yo puse pero entonces se mueren ahí adentro. Ellos salen de las cañas. Fuera yo lo agarrara porque muchos grande que andan.

-Yo tengo una vecina que sale, ahí un ratón, un ratón. Corriéndole jejeje.

-Yo lo paro así y son grande.

-Si grande.

-Los grande no hacen daño son los chiquitos. Los chiquitos son los dañinos. Pero yo he encontrado en el almario paria.

-Y yo he encontrado ratones en el gavetero también. Paria, paria.

-Si esos rajieritos.

**¿Y porque hay muchos ratones?**

-Los ratones son por la caña, eso es por la caña. A veces es por la caña y a veces por la basura también, porque la basura llama ratones.

-Claro la basura llaman ratones.

**¿Y qué hacen la gente para quitar los ratones, hacen o no hacen algo?**

-Lo que hacemos es que fumigamos. También compramos venenos y la ponemos en las cosas donde ellos se metan, a mí me gusta comprar de lo que se pegan porque es que a veces ellos se mueren y es una incomodidad porque uno no lo encuentra y eso es un bajo.

-De cual usted compra.

-Yo compro de la cosa que se pega a vece o si no le pongo veneno para su suerte.

-Y cuales son eso que usted compra.

-De lo que se pega.

-A pues yo tendré que comprar dona porque…

-Pero hay un hombre que trae todos los sábados a treinta pesos.

-A veinte cinco pesos.

-Ay, pero yo por matarlo doy hasta cuarenta eso pájaros me tienen al volar.

-La última vez que yo puse allá yo me arrepentí, porque se guardaron en la cueva y algunos adentro de la casa con el bajo yo tenía que estar con el abanico prendido por todos los lados. Hasta en la estufa se esconden. Y afuera en el patio.

-Si, es incómodo cuando uno le pone veneno y se guardan.

-Yo le echo mucha tierra a la cueva.

-Yo lo que le eche fue agua caliente con suavitel.

-Después que tú le eches eso tú le echas tierra.

-El dueño dijo que iba a ir a fumigar pero…

-O si no tú lo cierras con cemento.

-Hablen con Clemente, ya él no vive ahí en ese barrio?

-No el ya no vive en ese barrio, pero él no trabaja en sanidad.

**¿Usted iba a decir algo?**

-Allá casi no ponemos venenos por los niños. Si a ellos lo matan, claro porque si mata los ratones.

**¿Pero le ha pasado a ustedes?**

-Ay no, gracias al señor, por eso yo no lo pongo porque el mío todo lo que ve se lo mete para la boca.

-Así también la mía todo lo que ve se lo mete en la boca.

-Yo cuando lo pongo, me levanto bien temprano, ya a la cinco estoy de pies recogiendo lo mío, y mira ella ha dejado eso un poco ya.

**¿Entonces algo más de los ratones?**

-Ay no, ya no tengo más que decir.

**Entonces ustedes dijeron que eso viene por las cañas o la basura ¿Quiero escuchar más del asunto de la basura que está en [este barrio], que pasa con esa basura, cuéntenme un poco más de eso.**

-Es que a veces el camión de la basura no viene el sábado y tiene uno que esperar el otro sábado. Entonces la gente tira la basura para las cañas.

-Verdad uno la tira la basura. Porque uno no puede tener un saco de basura ahí ocho días. Allí hay una gente que viven pegada de la calle verdad y ellos tiran el desperdicio en la calle y después ese mal olor.

**¿Entonces como la gente se hace con la basura--ustedes en tiran en la caña cuando no viene el camión?**

-Exactamente.

**¿Y cuáles son las otras opciones?**

-También podemos, si chocan un perro hay adelante tenemos la mana que de una vez lo tiraos para hay atrás y entonces el mal olor nos da a nosotros.

**Ok, porque viven cerca de la carretera, entonces todo lo que está en la carretera está cerca de ustedes, ¿mucha gente quema basura?**

-Sí.

-Sí.

**¿Cuándo no viene el camión es muy difícil aguantarlo?**

-Claro, entonces hay que quemar la basura por no botarla o tirarla para la caña, pero cuando la queman ahí eso también nos afecta mucho a nosotros y a los niños, si y a uno mismo también le hace daño. Porque el humo viene contaminado.

-Eso viene muy contaminado, como dice ella y nos hace mucho daño.

**¿Algo más del asunto de la basura, es un problema grande o no es muy común, que piensan ustedes?**

-Bueno, la basura es un problema grande porque uno no puede estar con la basura, obligado ahí que deshacerse de la basura. Eso tiene mucha contaminación.

-Yo la entro en funda y la entro en saco y cuando no la tiro. Porque no me voy a quedar con la basura.

-Yo la echo en fundita, yo traigo mucha funda.

-Yo la echo en funda y la entro en un saco.

-Yo las de cocina la echo en una funda y la engancho.

-Por eso está tan feo porque echan la basura para la calle.

-La mayoría porque yo la meto en funda y después en saco, la mayoría pero cositas así de la cocina yo la tiro para allá atrás. Uno lo que echa en la basura grande es papeles.

-Pero yo no la tiro en el basurero porque yo no tengo patio y el pedacito mío es al frente, y lo que yo consumo más es basurita de compra y disparaticos.

-Yo también de la compra y los papeles del baño que uno siempre lo mete en funda y es mejor meterla en saco.

-Los pampers lo que tienen muchachos chiquitos.

-Bueno ya cuando viene el camión de la basura, yo si he contaminado mucho porque uno la tira para allá.

-Lo pampers lo que yo hago es que lo entro en funda y lo amaro y la pongo en una área aparte.

-Yo estoy cansada de los pampers porque por allá por mi casa hay un hombre al frente y parece que ellos tiran la basura y tiran los pampers para el frente, hubo uno que los perros lo cogieron y se lo llevaron a la vecina mía. Jejeje.

-Si lo perros sufren de eso de sacar la basura y trasladarla para lo patio ajenos.

-También es duro cuando uno envasa sus pampers en un lugar y lo engancha y entonces otra persona lo tiran así como que es un papel normal. O un lujo y eso es una asquerosidad que eso da, mira… a veces los tiran cerca y los perros van y se lo traen para la casa a uno.

-Yo lo agarro y lo engancho en una mata.

-Y uno normal imagínate uno va y lo recoge porque que uno puede hacer. Cuando pase el camión.

-Allá, uno no ve eso de Pampers, porque todo aquel que tiene su muchacho chiquito y son muy cuidadoso.

-Cuando yo tengo mucha basura y el camión no pasa, yo le pago a un moto concho para que me la bote. Y esa pupú hiede mucho.

-Ese terreno que está a dos casas de tu casas ¿Viven por ahí la gente?

-No, Me dicen que la dueña es la hermana de Raquel la que tiene el colmado, eso lo tienen de vertedero y mucha humedad hay ahí.

-Sí, ahí fue donde yo busque dos hombres y les pague para que me quitaran el agua que tenía en el frente, cuando tú me dijiste:¿hay que ver que se hace con esto aquí?

-Si tú lo arreglaste.

-Sí, y me metí de atrevida a un patio ajeno y ahí tape todos los tubos que estaban rotos que de ahí era que venía toda esa agua y tú ves cómo esta esto, resolvimos y toda esa agua era de ahí que salía y que se acumulaba.

-Si porque eso te está haciendo efecto.

-Si, que mandaba agua como sin control y yo busque dos hombres y les pague y lo mande a tapar. El día que ellos vengan y yo salga de aquí, y yo salga, bueno lo mande a tapar, porque había problemas y reclamo, pero yo fui quien mando a tapar eso ahí, porque uno se puede cruzar donde uno vive [ella], porque después uno esta cruzado.

**¿Hay personas que coleccionan vidrios o plásticos para reciclar o para vender?**

-¿Ya no hay botellero?

-A bueno, el del otro lado de la calle.

-Por mi casa hay una señora que recoge botellas y las junta ahí.

-¿Ella la compra?

-No, la vende donde Juan, donde cruzan los muchachitos y venden cuando salen de la escuela.

-¿Y en el mismo barrio nadie compra?

-No, del otro de donde Juan es que compran hierro, cosas plásticas, pero del otro lado. A la vera (al lado) del cementerio ahí.

**Podemos hablar un poco de las aguas, de las tuberías o de los problemas con esto, quiero escuchar eso, algunos mencionaron, que a veces hay tuberías rotas. ¿Qué pasa con eso, eso trae problemas o hay un proceso para arreglarlo?**

-No, porque cuando allá se rompe una, nosotros la arreglamos. Nosotros lo arreglamos en el pedazo. Porque a nosotros es que nos afecta y tenemos que arreglarlo obligado y eso es muy raro.

**Es muy raro, algunos mencionaron que no viene el agua por mucho tiempo, ¿Qué hacen en este caso?**

-Eso es muy raro. Si no hay agua, buscarla en otro lado, un día así y es muy raro, que tengamos que buscarla en otro lado el agua.

-Envían un chin y otro día, pero durar tanto tiempo sin agua, no, no gracias a Dios, no estamos sufriendo de eso aquí en Consuelo. Todos los días mandan agua, es muy raro que no manden agua.

-En antes si, pero si hay una tubería rota, se puede durar un día o dos, pero después que la arreglan todos los días la mandan. Yo no sé si por allá sube igual, pero aquí sube mucha agua.

**¿Y la luz es un problema?**

-La luz es un caos. Usted sabe, la luz no anda parando aquí se va cuando tú estás pensando de que habrá luz, jejeje totalmente lo de la luz es un caos.

-Lo de la luz es de más problemas que con el agua, porque el agua la mandan diario, es muy raro que no manden agua, si hay algo roto no la mandan, porque hay que arreglarlo, pero con la luz sí, porque la luz tiene su horario lo normal porque no la tenemos fija.

**¿Otras experiencias con esto? Cómo es la vida comunitaria en [el barrio], o sea ustedes viven en comunidad o cada quien tiene su ladito, hacen comunidad todos desde aquí y hasta allá?**

-No, porque ahí arriba hay un terreno que yo no lo conozco. Y yo vivo en Buena Vista.

-Y yo tengo casi dieciséis años aquí.Y nosotros vivimos ahí mismo.

-A ella yo como que la he visto, porque vive ahí mismo ¿Usted vivía por allá?

-Sí, yo vivía por allá. Yo como que la he visto a usted, pero no recuerdo bien pero de ninguna de ella las he visto.

-De allá arriba conozco a muchos y de allí conozco a unos cuantos, porque yo estoy aquí desde los nueve años.

**¿Y la junta de vecinos trabaja en el barrio entero?**

-¿Y allá hay de eso?

-Allá arriba es que tienen junta de vecinos y allá abajo donde Yovanny.

-Allá abajo hay dos juntas, allá abajo esta Yovanny y aquí arriba…

-¿Qué Yovanny?

-Yovanny el de allá arriba el del barrio, ¿No es el que tiene la junta de vecinos?

-Yovanny es el del otro barrio.

-Nosotros teníamos junta de vecino, cuando era Iris la que estaba en eso.

-Allá arriba Iris y allá abajo Yovanny.

-Sonia era la presidenta de la junta de vecinos.

-¿Qué Sonia?

-Sonia, la hija de Digna.

-Ella no hace nada por la junta de vecinos y si hacen algo. Y si hacen algo mínimo, hay que estar visitándolos a ella y haciendo reuniones a su gente, porque a nosotros no nos buscan, tu vez ella siempre cuando iba a hacer algo siempre nos llamaba. Aquí estamos huérfanos en [nuestro barrio].

-Iris sí, pero aquí no hay junta de vecinos, solo se hace reunión cuando es para hablar de la tarjeta hay sí.

-Es decir, cuando hay cosas que nos afecta mucho en la comunidad, que hay que reunirse un grupo a ir un tal equipo, hay Iris esta de pies y todo el mundo parado. Si es con la luz es un lio. En el pedazo de nosotros nadie se junta, cuando se daña algo nadie quiere y quienes somos lo que nos paramos ahí a hacer la lucha, el grupo de Iris y el barrio de allá a los que nos afectan nosotros queremos una persona responsable porque cuando tu necesita ahí es que tu tiene que ver.

-Estamos hasta huérfanos nosotros. A nosotros no iban a mandar hacer la callecita.

-La de allá atrás verdad. La de allá atrás no la iban a hacer y se opusieron supuestamente lo de la junta de vecinos de aquí arriba.

-Tono fue uno de lo que se opuso y allá abajo se opuso Judit.

-Nosotros nos pusimos contento porque iban a ser la carretera de aquel lado.

-Según yo oigo fueron esas dos personas que se opusieron.

-Nosotros siempre tenemos que sacar el muerto de aquel lado.

-Entonces eso es algo que nos favorece a todos verdad, y no afecta también a todos porque uno no va a estar en la callecita del peatonal, porque se opusieron supuestamente por lo motores que iban a pasar y que teníamos muchos niños, y antes cuando teníamos la máquina, tu nunca oíste que no mataron un niño aquí en este pedazo, del lado de allá si mataron un niño. Y de este lado nunca mataron a nadie y se opusieron, entonces estamos huérfanos de eso, el síndico mando a medir cuando iba hacer la calle y la mayoría de gente de aquí arriba lo que dejaron fue un callejoncito entonces con ese callejoncito nos podían tirar la calle, porque somos brutos, necesitamos la callecita para tener más higiene en el barrio, no te meta adelante, verdad, mejor coja de la de atrás y no de la de adelante, entonces cogieron los brutos cogieron los frentes.

-Ah, donde estaba la línea.

-Sí. Lo que hicieron fue que en vez de coger para allá se tiraron más para adelante, entonces quien se hace daño, nosotros mismos, nos hacemos daño, porque mira a donde está la empalizada mía, hay mucha gente que me han dicho pero échate para adelante, no ella se va a quedar ahí, porque si algún día el síndico viene y me la quita y viene a mí no me la va a tumbar--al contrario, tumbe una mata de jobo que había en mi casa yo dije bueno si el viene y esto se da y él tiene que coger hasta donde está la mata de jobo y tumbarla la tumbo y la de palma que tu vez que yo tengo ahí también la tumbo porque nosotros somos lo que necesitamos que no limpien nuestro frentes y que nos ayuden en el barrio con nuestra comunidad, pero si nosotros mismo no ponemos como lo van hacer.

-A mí me dijeron que echara más para atrás y yo dije que no, que la voy a dejar ahí.

-Lo mío se queda ahí. Porque si vienen a hacer la carretera entonces no podemos dejarla ahí.

-Para mí que hacer una calle atrás para mí es un peligro, porque por esa carretera hay muchos niños, entonces no nos conviene.

-Cada quien tiene que cuidar su muchachos.

-Pero antes cuando teníamos la maquina ustedes que somos vieja en la carretera. Sí, que la maquina ni dejaba dormir a uno.

-Y estábamos al pendiente porque no podemos estarlo ahora con los motores.

-El que tiene su hijo realengo es el que tiene que estar asustado, pero usted que tiene su muchacho domado…Pero yo digo que si antes lo cuidábamos del tren que era más peligroso. Y peor. Porque ahora no podemos cuidarlo del motor, lo que tenemos es que ponernos de acuerdo. Y tener las casas cerradas. Verdad, no ponemos de acuerdo y no mandan a tirar pal de policías acostados para la callecita peatonal y estamos más en la higiene, pero muchos de brutos lo que hacen es que tiran las casas más para adelante, disque por los muchachos pero y ante cuando teníamos el tren, porque yo vivo aquí desde los nueve años. Desde los nueve años yo vivo ahí en la carretera.

-Pero mira lo que yo tuve que hacer allá en el patio frente a la casa.

-Del otro lado mataron un niño y de este lado nunca lo han matado gracias al señor. Oíste yo tuve que hacer dos policía acostado y compra dos funda de cemento para hacerlo, porque por todo estos muchachos jugando y pasaban los moto conchos vuelto al pecado y hice los dos policías acostado y ahora pasan mirando.

-Pero eso era lo que teníamos que hacer, reunirnos un grupo allá arriba. Ya entiendo porque por ahí por donde vivimos entra carro y de todo vehículos ahí.

-Vamos a reunir todos después que no hagan la callecita peatonal, como madre y padre vamos a reunir todos y el que no pueda reunir para la funda de cemento, pon una que yo pongo una y con la arena pon un granito tú, un granito tú y otro tú y vamos a echar pal den policía acostado si es por lo motores pero eso ellos ya pusieron y por esa son las cosas que se no llenan los frentes de agua y de cualquier tubo roto que haya, entienden--todo es porque no compartimos ideas que es lo que tu dice.

**Gracias a ustedes por compartir, de esto yo he aprendido mucho escuchando de esto quiero escuchar más ideas que tenga que ver con lo que discutimos, pensando en estos problemas, que podemos hacer juntos como dentro de la comunidad para mejorar algunos de esos problemas? ¿Que ideas tienen ustedes? Quiero escuchar de sus ideas y de lo que hemos discutido.**

-Bueno, tratar de respetar la basura, hay que acumular menos basura y menos agua porque son cosas que a uno mismo es que le afectan.

**¿Y cómo podemos acumular meno basura, han pensado en una idea para disminuir la cantidad de basura?**

-Bueno eso es un lio. Para uno acumular menos basura es como yo le digo, yo tengo un saco con una funda y la basurita yo la voy echando ahí entonces cuando hay bastante ya yo lo que hago que la amaro y la meto en saco.

-Es así porque donde yo vivo, tu ves, como yo vivo, entonces como acumula basura, como tengo basurero?

-Pero no yo que tengo patio me gusta que la basura se acumule, desde que yo me levanto bebo café y de una vez a barrer e mas por ahí están todos los patios limpios.

**¿Ustedes piensan que sus vecinos están falta de conocimientos sobre la basura y como le puede hacer daño a la salud de la gente?**

-Claro que deben saber que le hace daño a la salud toda esa basura.

**¿Pero saben eso sus vecinos o no entienden eso?**

-Ellos lo entienden porque por eso es que se mantienen limpiándolo, osea eso es allá en el pedazo de nosotros casi no se ve eso de basurero en el medio, no te sé decir en el barrio porque yo te hablo del pedazo donde vivo.

-Para allá abajo se ve más porque hay unos cuantos solares vacíos y usted sabe que cuando los gatos están amarado los ratones están de su cuenta, si el dueño no está pendiente ahí va todo el mundo a tirar, por ejemplo: mira la casa a la que tú me hiciste la pregunta la mayoría de gente que viene de allá atrás con la basura, ¿qué hace? que cuando el camión no pasa, dejan los sacos ahí y ahí se acumulan para ese criadero, cuando no cortan la hierba rápido y toda esa basura los perros la riegan ¿qué es lo que se forma? un criadero de ratones y cucarachas y todo eso ¿para dónde va? Para donde uno, donde uno limpia, ¿entiende?

**Entonces pensando en otras maneras para reducir la basura, a veces puede ser difícil hacer algo si sus vecinos no están también involucrados ¿Qué ustedes opinan de eso, si ustedes podría cambiar algo, seria apoyo de eso de los demás? Sería difícil motivar a los demás?**

-Bueno, en eso es un caso, es un lio, uno quisiera que si uno tiene su patio limpio en su casa a uno le gustaría que el vecino también lo tenga, y es un lio persuadir al vecino a que haga lo mismo ¿entiende? Es un lio, porque hay personas que somos ñoño de más con eso, es decir, que si tienen un saco de basura mal puesto y yo voy y le digo: vecino tiene ese saco de basura mal puesto ahí, se pueden ofender por eso, ¿entiende? Es un lio como quiera.

**¿Cuáles experiencias han tenido los demás, hay otros esfuerzos comunitarios que a lo mejor sería muy sencillo pero que pueda mejorar algo la calidad de los hijos o del agua, otras que no hemos discutido? Yo sé que estos temas son muy difíciles y que si tuvieran respuestas muy fáciles lo podrían entender…bueno, ustedes compartieron mucho yo creo, entonces muchas de sus experiencias de lo que ustedes observan y realmente son muy importante para nosotros y gracias por compartir sus ideas con nosotros, ustedes compartieron y vamos analizar esa conversación y buscar lo que podemos y hacer proyectos de lo que podemos hacer en la comunidad con ustedes ¿Hay alguno que otro quiera mencionar otro video o alguna otra preocupación?**

-Ya no hay más nada. Yo misma no tengo más nada.

**¿Ustedes creen que sea posible o sea beneficioso que le hagan una peatonal al barrio, están de acuerdo?**

-Claro, claro. Usted no sabe el cambio que eso va a dar.

-Usted sabe que cuando hacen peatonal o algo abren…. En Cachipero hicieron eso y la gente lo aceptaron pero Cachipero es un barrio amplio, pero aquí hay casas que están muy cerca y hay gente que no van a querer y principalmente….

-Pienso yo que las gente que estén interesadas deberían unirse, van al ayuntamiento, porque yo creo que hay proyectos de continuar asfaltando y pueden presentar su propuesta a través de una carta o un grupo que vaya.

-Que la tiren donde la tiraban antes dónde dejaron lo estrecho, que lo que fue que pensaron en su propia conveniencia según ellos y que se lo dejen así y que la tiren hasta donde ellos puedan ¿Verdad? porque el que cerró era porque no quería calle y quería vivir en el fango ¿Verdad?

-Aquí es por el hoyo.

-Por el frente de tu casa ellos pasan porque pasa una guagua allá sí.

-Yo digo que eso es brutalidad ¿saben porque? porque una casa de esas coge fuego y ¿Por dónde va a entrar el camión? A eso es a lo que me refiero hace rato, que no se si al tocar el tema alguien se pueda sentir mal ¿entiende? Pero es algo que nos afecta a todos, porque si entran, un camión entra nosotros tenemos que estar comprando materiales, si no hay espacio por donde entrar el camión ¿Por dónde nos no la van a llevar? No hay espacio ¿entiende? Si hay un fuego nos vamos todos, porque no hay por donde cruzar el camión, bueno tú has caminado el área de nosotros tú más o menos ves ¿hacia dónde puede llegar el camión?

-Por allá entra el camión. Por la línea.

-Por la medio si, la gente que vivía ahí dejo eso igual.

-No para allá eso está mejor pero aquí arriba ahí un pedazo que no puede hacerla.

-Yo he pasado por una arte que esta como de ese ancho.

-Si hubiesen cogido desde allá desde la entrada del cementerio que tú sabes que eso es una callecita hasta la salida de aquí de la bomba, tú sabes lo linda que había estado esa callecita. Precioso se hubiese puesto el barrio, porque cambia tiene otro cambio y tiene otra luz, pero como lo vamos hacer.

-Por allá estamos igual, el ayuntamiento cuando ellos van.

-No porque las casas de por tu casa están igual todas, aunque le den algún dinero o algo porque en Cachipero paso así, aunque no querían la abrieron.

-Pero te digo algo. Se ve mejor ahora. Se ve mejor y deberían de hacerlo aunque le devuelvan a la gente lo que invirtieron porque es que será otro cambio y se verá diferente que tenga entrada y que tenga salida, pero ahora tiene entrada y no tiene salida porque por donde debería de estar la salida lo que dejaron fue callejones.

-Lo que yo digo que si se muere una gente del otro lado es que tenemos que sacarlo. Si del lado atrás porque no tenemos por donde sacarlo. Pero yo he hablado con una cuantas de los de allá.

-Tu sabes el cambio que se haría.

-Pero es que en barrio peores que el de nosotros tu vez la callecitas nítidas y uno desde que cae un chin de agua no tiene por donde caminar, del lodo y del agua aposada y eso lo que trae es contaminación, porque dizque los motores, pero y la maquina cuando cruzaba que era un tren con que se yo cuanto vagones, gracias a Dios que no quitaron eso, pero como quiera a vemos gente que somos… Mira yo no estudie y hay gente que son brutos de más, que dizque el motor que los muchachos pero cuando en la carretera no ha visto una balsa de niños toda la vida y todo el mundo escuchaba el tren porque el tren pita digo pitaba y cada quien se mandaba y recogía a su muchachito y cuando pasaba eran libre de nuevo es verdad eso era lo que decíamos somos libre de nuevo pero cuando pitaba aquí allá abajo y recogía a su muchachito y o guardaba y cuando pasaba ya somos libre de nuevo, y de noche no podíamos dormir tampoco por la bulla del tren.

-Y todo eso está lleno de casas.

-Hasta se voceaban por allá, ahí viene la maquina eso sonaba que la cama hacia tututu.

**Una pregunta más ¿ustedes creen que las personas que viven aquí tienen responsabilidad para mejorar su barrio, y la otra pegunta es, si sienten poder de hacerlo o no? Que si ustedes como comunidad tienen algún grado de responsabilidad de que yo voy a cambiar por eso, yo voy a luchar porque mi barrio sea diferente por la cosa que no están afectando para luchar todos unidos.**

-Bueno, si todos pensaran como yo o igual fuera muy diferente. Fuera muy diferente, porque es que a veces, mira ahí hay unos cuantos muchachos que recogieron una cuantas firmas y no se en que paro eso, si fue que se llevaron de los otros que se oponían a que fue, hasta mi hermano fue y hablo con el síndico respecto a eso y él dijo que sí que eso estaba en proyecto pero hasta ahora no hemos visto nada.

**¿Qué piensa los demás de eso?**

-Pero si todos no pusiéramos de acuerdo ya hubiesen hecho esa callecita y otras cosas más porque esos desagüe también tu sabes la veces que han ido cuando están en política buscando votos y que sí que no van a hacer el desagüe y después que suben se olvidan de los desagüe y de todo las promesas que le hacen a uno, si pero eso es a cada rato, promesas y más promesas que los desagüe y que no van a ayudar con la callecita y cuando suben se olvidan de uno.

-O si no van y hacen un aguaje.

-Un aguaje y ya. Y se van y no vuelven más.

-Pero después este barrio se va a ver diferente si ese proyecto se da porque lo limpio es limpio.

-Ellos empezaron dizque que iban a limpiar todas las casas y que no la iban a arreglar. Y mentira no arreglaron nada.

-Nos cogieron los cuartos.

-Si allá fuero también, yo estaba metía en eso también.

-Por ahí no cogieron lo cuarto usted. Jejeje. Jejeje.

-Reunión y reunión y nunca la arreglaron. Reunión por todos los lados.

-El de barrio Cachipero él le vendió sueños a la gente, dizque que iba a construir las casa, usted se acuerda de un proyecto en santo domingo que decía un barrio para mi país o una casa con mi país. Que era un grupo de jóvenes. Eran perfecciónales de la construcción entonces el vino aquí presentando más o menos un proyecto igual pero él dijo que la personas que se inscribieran en el listado tenían que darle a él, no eran ciento veinticinco pesos u ochenta algo así.

-Yo gaste más. Jejeje.

-Y él recogió mucho dinero y dizque trajo siempre. Si el dinero es dizque para la foto, y la foto, el carnet ni el nunca aparecieron y todavía lo estamos esperando. A mí me cogió mi cuarto…

-Pero por ahí por la explosión estaban metiendo otra trampa, para que todo el mundo fuera otra vez con más cuarto, yo le dije vallan ustedes que yo estoy ocupada. Jejeje.

-Yo le digo vayan ustedes que yo no sé firmar. Yo no estoy en eso cuando Dios me ayude yo termino, mi hija peleando mami ojala te engañen y así mismo me engañaron y la difunta profesora me llama ven Sara a ver si te arreglan tu casa y yo fue huyendo dice mi hija ojala te engañen y me engañaron de verdad.

-Como una vez.

-Ya yo no caigo en esa no.

-Es que hay muchas trampas. Cuando yo viva a donde mi papa llego una señora. Miren ustedes van a participar en unos quince yo quiero que ustedes me den la ropa la suerte fue que la casa mía estaba cerrada con candado y la otra prima mía le dio toda la ropa y cuando vino la mama y donde está la mujer y se desapareció la mujer con todo y la ropa y o volvió jamás ni la placa le vimos.

-Yo no caigo en gancho así, yo caí en ese porque imagínate porque fue con todos os datos la cedula todo. Jejeje.

-Si dizque por la necesidad de uno superarse y de vivir mejor a veces uno entra en casa. No, pero como nosotros vimos que era de la Primera Dama y como ella arreglo en El Seibó. Es verdad que no mencionamos La Primera Dama. Como ella arreglo en El Seibo verdad porque vimos todas las casitas que ella hizo en El Seibó, nosotros estábamos contando con que no iban a arreglar la casita.

-Jejeje. Y donde están en vez de arreglarla nos engañaron, es que el sueño pero que raro porque uno duerme de día, eso es porque está ahí. ¡Ay Dios mio!

**¿Bueno pues algo más que compartir?**

-Ya. Ya yo no tengo más nada.

**¡Otra vez muchísimas gracias!**
